# Modeling the asymmetric evolution of a mouse and rat-specific microRNA gene cluster intron 10 of the Sfmbt2 gene

**DOI:** 10.1186/1471-2164-12-257

**Published:** 2011-05-23

**Authors:** Stefan Lehnert, Vladimir Kapitonov, Pushpike J Thilakarathne, Frans C Schuit

**Affiliations:** 1 Gene Expression Unit, Department of Molecular Cell Biology, Katholieke Universiteit Leuven; 2 Genetic Information Research Institute, 1925 Landings Dr, Mountain View, CA 94043, USA; 3 Interuniversity Institute for Biostatistics and statistical Bioinformatics, Katholieke Universiteit Leuven, Kapucijnenvoer 35, Blok D, bus 7001, B3000 Leuven, Belgium, and Universiteit Hasselt, Belgium

**Keywords:** microRNA, miRNA, simple repeat, SINE B1F3, evolution, gene conversion

## Abstract

**Background:**

The total number of miRNA genes in a genome, expression of which is responsible for the miRNA repertoire of an organism, is not precisely known. Moreover, the question of how new miRNA genes arise during evolution is incompletely understood. Recent data in humans and opossum indicate that retrotranspons of the class of short interspersed nuclear elements have contributed to the growth of microRNA gene clusters.

**Method:**

We studied a large miRNA gene cluster in intron 10 of the mouse Sfmbt2 gene using bioinformatic tools.

**Results:**

Mice and rats are unique to harbor a 55-65 Kb DNA sequence in intron 10 of the Sfmbt2 gene. This intronic region is rich in regularly repeated B1 retrotransposons together with inverted self-complementary CA/TG microsatellites. The smallest repeats unit, called MSHORT1 in the mouse, was duplicated 9 times in a tandem head-to-tail array to form 2.5 Kb MLONG1 units. The center of the mouse miRNA gene cluster consists of 13 copies of MLONG1. BLAST analysis of MSHORT1 in the mouse shows that the repeat unit is unique for intron 10 of the Sfmbt2 gene and suggest a dual phase model for growth of the miRNA gene cluster: arrangment of 10 MSHORT1 units into MLONG1 and further duplication of 13 head-to-tail MLONG1 units in the center of the miRNA gene cluster. Rats have a similar arrangment of repeat units in intron 10 of the Sfmbt2 gene. The discrepancy between 65 miRNA genes in the mouse cluster as compared to only 1 miRNA gene in the corresponding rat repeat cluster is ascribed to sequence differences between MSHORT1 and RSHORT1 that result in lateral-shifted, less-stable miRNA precursor hairpins for RSHORT1.

**Conclusion:**

Our data provides new evidence for the emerging concept that lineage-specific retroposons have played an important role in the birth of new miRNA genes during evolution. The large difference in the number of miRNA genes in two closely related species (65 versus 1, mice versus rats) indicates that this species-specific evolution can be a rapid process.

## Background

Micro RNAs (miRNA's) are 19 to 22 nt long, non-coding, single-stranded RNAs that can fine-tune the expression of protein-encoding genes [1,2]. One example is the post-transcriptional repression of mRNA targets involving the so called miRNA "seed" which is nt 2-8 of the mature miRNA which recognizes complementary bases in the 3'untranslated region of the mRNA target [3]. miRNA genes form primary transcripts that are converted by Drosha to miRNA precursors of 70-90 nt length. Processing of miRNA precursors into mature miRNA's is catalyzed by the RNA processing enzyme Dicer [4,5]. Processing enzymes recognize the secondary hairpin-structure of the miRNA precursor [6]. Most miRNA precursors have indeed a distinctive stem-loop structure that is often highly conserved among distant species and that is used to distinguish them from other small RNA classes.

The exact number of miRNA genes, collective expression of which makes the miRNA repertoire of an organism, is not known and the question how new miRNA genes arise is an interesting and insufficiently studied problem in evolutionary biology. The conserved hairpin structure in miRNA precursors was applied in two primary miRNA identification methods: directional cloning and computational identification [7,8]. Consequently, newly evolved, not-conserved miRNAs were likely to be overlooked by these methods. Furthermore, some miRNA gene candidates accumulated sequence mutations that, over time, either led to mature miRNA genes or to gene inactivation [9]. Both actions make it difficult today to trace back the origin of miRNA genes. Using a deep sequencing approach, a large group of evolutionarily young miRNA genes was discovered in *Drosophila *[9]. In this study, a high birth rate of new miRNA genes was described (12 new miRNA genes per million years). The main sources for gaining miRNA genes in plants are based on miRNA gene duplication events and local inverted duplication events of short segments from protein-coding genes [10]. An alternative source of short inverted sequence segments could be based on transposons as these often carry terminal inverted repeats or as they can insert at short distance from their origin, resulting in an inverted gene arrangement [11]. The involvement of transposons in the birth of clusters of miRNA genes might be underestimated as computational miRNA detection methods were designed to exclude transposon sequences [8]. However, recent analyses in several mammalian species [8,12,13] indicated that a number of miRNA gene clusters were derived from repetitive elements. This may have contributed to "leaps" in the expansion of the miRNA repertoire in placental mammals [14].

Approximately, 40% of the mouse genome is composed of repetitive elements, the largest part of which is interspersed between or within genes. Two examples are simple repeats and B1 elements (short interspersed nuclear elements (SINE), an Alu-like family of retrotransposons in rodents). Each occupies a similar fraction of the mouse genome (2.3% vs. 2.7%) [15]. Mouse B1 elements are rodent-specific retrotransposons (length approximately 140-bp) that may have originated from reverse transcription of ancestral 7SL RNA [16]. Transposable elements like B1 in mice and Alu in humans were previously thought to be either harmless "junk" or to influence functional genes negatively [17]. More recently, however, it was proposed that retrotransposons can actually contribute to gene function and genome evolution, either by promoting local duplications, inversions or deletions events or by altering gene expression [18,19]. Simple repeats are present in micro- and, minisatellites which are short tandem repeats of one repetitive unit [20] with a characteristic length and sequence. For instance, in microsatellites, the repeat unit is 1 to 6 bp long. It has been shown that microsatellites are characterized by a high sequence mutation rate (10^-6 ^to 10^-2 ^per bp per generation) [21]. This makes microsatellites an adaptable reservoir for genetic changes, in addition to the phenomenon that numerous host genes evolved from retrotransposons [22,23].

A number of recent findings suggest that the biology of miRNA's and of retrotransposons may be connected at several levels. One starting point is the idea that RNA interference is a mechanism that protects genomes against an unacceptably high, self-destructive retrotransposon activity [24,25]. Interestingly, the RNA interference pathway and the maturation and action of miRNAs share some processing proteins and both mediate endonucleolytic cleavage [26-28]. Furthermore, the evolutionary growth of an Alu-rich primate miRNA cluster [29] and a marsupial miRNA cluster [30] were proposed to be driven by local duplication events involving retrotransposons. For the miRNA genes in the human chromosome 19 cluster, the encoded seed sequence was found to be complementary to the most conserved parts of Alu RNA [29].

In the present study we have analyzed the sequence of a large miRNA cluster which is specifically located in intron 10 of the Sfmbt2 gene on chromosome 2 of the mouse (from now on abbreviated as C2MC). This cluster is very rich in B1 retrotransposons and microsatellites, which are ordered in a regular manner. In rats the same repeat elements are present, but small changes in DNA sequence may have resulted in less-stable hairpins and failure to form mature miRNA from precursors. In all other mammals an ortholog of C2MC could not be detected. On basis of this analysis we propose that B1 retrotransposons have contributed to rapid growth of the mouse C2MC miRNA cluster, which further illustrates the connection between retrotransposition and the evolution of a miRNA repertoire in mammals.

## Results

### 1. Large mouse-specific miRNA gene cluster in intron 10 of the Sfmbt2 gene

**Figure 1:**
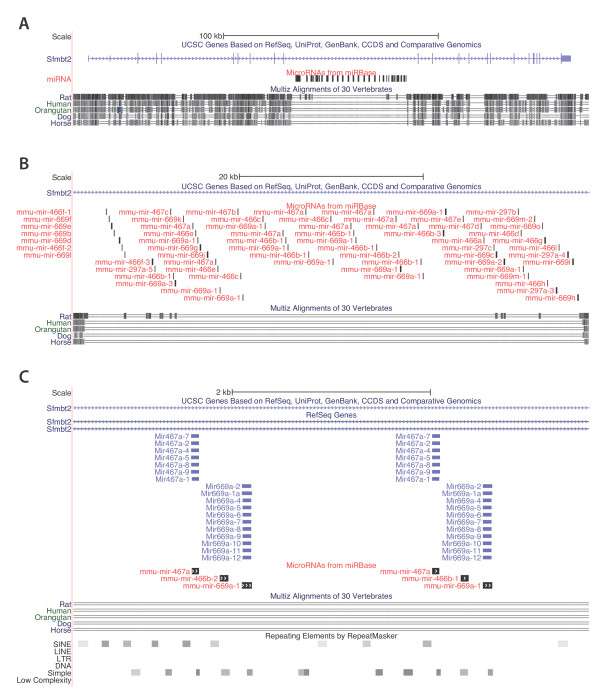
**C2MC is a large miRNA cluster in intron 10 of the Sfmbt2 gene**. Overview of the mouse Polycomb group gene *Sfmbt2 *is shown using the mouse UCSC genome browser (Version mm9 - http://genome.ucsc.edu). **A**) All miRNA genes are present inside a ~54 Kb region of *Sfmbt2 *intron 10, which is not conserved in other mammals except to some extent for rat (Chr2: 10,285,001-10,525,000). **B**) The cluster contains 65 miRNA genes and is flanked by conserved parts of intron 10 (Chr2: 10,385,001-10,441,000). **C) **The centrally located miRNA genes appear evenly spaced. The cluster's compartmentalized organization is indicated by multiple mappings of RefSeq Genes and by its repeating element content (Chr2: 10,418,831-10,424,030).

**Figure 2:**
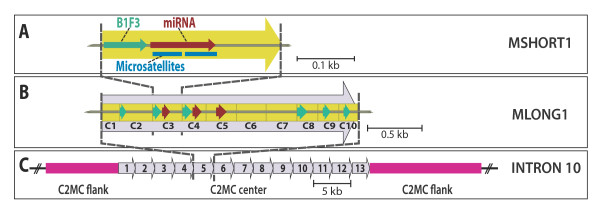
**Modular repeat composition of C2MC**. **A) **The basic repeat in C2MC is MSHORT1: this is a 296 nt unit, which contains a 75 nt 3' portion of a rodent B1F3 element and a ~83 nt pre-miRNA gene consensus, which overlapped with an annotation of two self-complementary microsatellites. **B) **An array of ten MSHORT1 copies (C1 to C10) forms a 2,5 Kb unit called MLONG1, which contains 3 miRNA genes (red) and 6 B1F3 elements (green). **C) **The center of C2MC is generated by tandem duplication of MLONG1 elements; the flanks of C2MC contain less regularly spaced MSHORT1 units (pink).

During our search for mammalian miRNA gene clusters in which SINE repeats may have contributed to cluster growth [29] our attention was drawn to C2MC, a large group of miRNA genes on chromosome 2 of the mouse. This cluster is located in intron 10 of the Sfmbt2 gene and spans more than 50 Kb (Figure 1A). The C2MC cluster is predicted to contain 65 miRNA genes which are annotated by UCSC and which are present in *miRBase*. Each miRNA gene in the cluster is encoded by the (+) strand, i.e. parallel to the coding strand of the Sfmbt2 gene. The C2MC cluster was found to be specifically present in the mouse when considering orthologs or paralogs of the Sfmbt2 gene. First, in or immediately around the paralogous mouse Sfmbt1 gene, no miRNA genes were found (Additional File 1 Figure S1A). Second, no miRNA genes associated with the Sfmbt2 gene were found in any other species, except for the rat where one miRNA gene was found in intron 10 (Additional File 1 Figure S1B). In fact, we noticed that in the total 300 Kb area of the mouse Sfmbt2 locus, sequence homology to other mammals (including rat) was lowest with the mouse intron 10 region (Figure 1A). Low sequence homology was also found with between mouse Sfmbt2 intron 10 and the same region in genomes of rabbit, ground squirrel, guinea pig and kangaroo rat (Additional File 2 Table S1). As can be seen in Additional File 1 Figure S1B, the rat orthologous intron 10 region on chromosome 17 is of the same size as in the mouse (about 60 Kb) and has a high density of simple repeats and SINE elements. Repeats are also clustered in intron 10 of the mouse (Additional File 1 Figure S1C). When we looked in further detail in the central region of intron 10 of the mouse (Figure 1C) we found a regular spacing of miRNA genes, simple repeats and SINE repeats with an apparent repeat length of 2.5 Kb and we propose to call this repeat unit MLONG1. A tandem array of 13 MLONG1 elements is sufficient to account for all miRNA genes in the center of the C2MC cluster (Figure 2). The SINE repeats of the mouse Sfmbt2 intron 10 are all classified as B1, which belong to the Alu family. The B1 elements in MLONG1 are more frequent, significantly smaller and more uniform in orientation than B1 elements in the collected set of all other introns of mouse chromosome 2 (Additional File 1 Figure S2). We next investigated the exact composition of the 2.5 KB repeat unit MLONG1.

### 2. Tandem repeats of MSHORT1 explain the cluster of miRNA genes in the Sfmbt2 gene

BLAST analysis of MLONG1 indicated that this repeat was in itself a composite unit, built by a tandem array of ten MSHORT1 elements (Additional File 1 Figure S3A). The MSHORT1 consensus sequence (296 nt) aligned to 193 individual MSHORT1 copies (Additional File 2 Table S2) that form a tandemly head-to-tail array in intron 10 of the Sfmbt2 gene and together account for >90% of the intronic space. A more detailed view of the MSHORT1 element consensus (Figure 2A) suggested that the first 75 nucleotides are a 3' fragment of the rodent B1F3 element, while the middle part (83 nt) was homologous to the pre-miRNA gene consensus which overlapped with two self-complementary microsatellites. The tandem repeat of MSHORT1 elements could account for all 65 pre-miRNA genes that are annotated in the cluster. When clustering ten MSHORT1 elements into a MLONG1 element, loss of sequence and accumulation of mutations may explain why only three pre-mRNA genes and up to seven B1F3 fragments are recognized by BLAST (Figure 2B). A similar situation exists in intron 10 of the Sfmbt2 gene of the rat. In this species, a 197 nt RSHORT1 consensus was found that aligns to 236 copies which are also tandemly repeated (Additional File 1 Figure S3B, Additional File 2 Table S3). The MSHORT1 and RSHORT1 consensus elements were found to be homologous to each other (Additional File 1 Figure S3C), but MSHORT was extended by 0.1 Kb at its 3' end.

### 3. The mouse-specific miRNA gene cluster expanded in two phases

**Figure 3:**
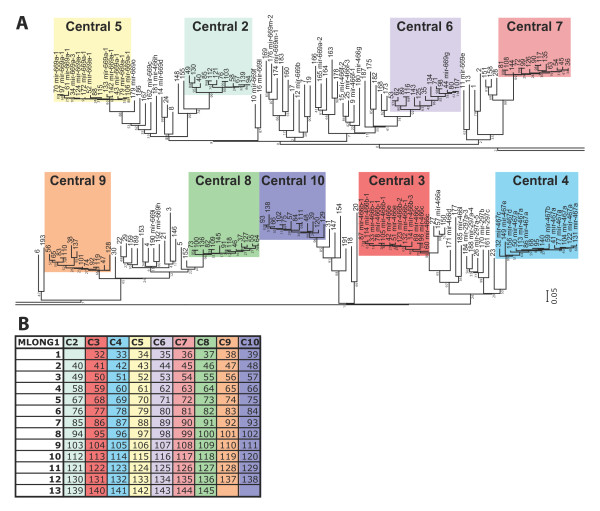
**Evolutionary relationship of the MSHORT1 elements in C2MC**. **A) **The phylogenetic tree of all 193 MSHORT1 elements that are present in C2MC is shown. MSHORT1 copies are numbered from left to right for their location in C2MC. When a miRNA gene is present in the MSHORT, it is indicated as well. MSHORT1 copies in the central region of C2MC (see Figure 2C) are colored, while the MSHORT1 copies in two C2MC flanks are white. Branches corresponding to partitions reproduced in less than 50% bootstrap replicates are collapsed. The percentage of replicate trees in which the associated taxa clustered together is shown next to the branches. The scale bar represents 5% of sequence divergence. **B) **The sequential order of MSHORT1 copies is highlighted. The MSHORT1 units that belong to the same sub-cluster are listed in columns. Rows show the arrangement of MSHORT1 units in the corresponding MLONG1 copies. Please note that the first 31 MSHORT1 copies are located in the left flank of C2MC (Figure 2C), while copies 146-193 are in the right flank.

We next constructed the phylogeny the individual MSHORT1 elements in order to model the evolutionary growth of C2MC. BLAST analysis allowed the identification of 9 MSHORT1 sub-clusters each of which containing 12 or 13 copies of highly similar MSHORT1 elements (highlighted by the nine different colors in Figure 3A). MSHORT1 elements that could not be sub-clustered (white in Figure 3A), were more distant from each other, as were MSHORT1 elements compared across sub-clusters. All sub-clustered MSHORT1 copies were found to be located in the center of C2MC (Figure 2 and 3B), while the MSHORT1 elements that could not be sub-clustered were specifically present in the C2MC flanks. The regularly ordered arrangement of MSHORT1 units could be modeled by the formation of MLONG1, which is tandemly repeated in the center of C2MC (Figure 2 and 3B). Similar observations were made for rat RSHORT1 copies. However, the rat cluster central region has a less stringent fixation of RSHORT1 copies in longer units that would represent the rat equivalent to mouse MLONG1 (Additional File 1 Figure S4). These results suggest that, during the most recent expansion of the mouse miRNA gene cluster, which occurred in the C2MC central region, an array of tandem repeated MSHORT1 copies, fixed as MLONG1, was duplicated 12 times.

### 4. miRNAs in MSHORT1 and RSHORT1 are different from miRNAs outside the Sfmbt2 locus

All miRNA genes in MSHORT1 and RSHORT1 belong to miRNA families 466 and 467. A hallmark of miRNAs embedded in MSHORT1 and RSHORT1 is that the hairpin precursors overlap with two self-complementary microsatellites (AC)n and (GT)n, which might have shaped an ancestral hairpin. miRNAs of families 466 and 467 are not exclusively found within C2MC, but also on other mouse chromosomes and in other species than mouse and rat. However, BLAST analysis of other genomes (human, chicken, *D. melanogaster*, *C. elegans*) for MSHORT1 retrieved no results while in mouse and rat gave only results within intron 10 of the Sfmbt2 gene. To analyze the relationship between the members of the miRNA families 466 and 467 in detail, we selected the 10 pre-miRNAs of these two families that are located in other species and outside C2MC. We added 100nt up- and downstream sequence to evaluate cross-species conservation and compared the sequences to MSHORT1 and RSHORT1. All analyzed pre-miRNA sequences of the other species contained microsatellites resembling a (AT)n repeat instead of a (AC)n or (GT)n repeats as found in the C2MC miRNA genes. An exception might be the sequence containing has-mir-466 with (TATG)n and (CA)n microsatellites (Additional File 2 Table S4A). We also assessed the impact of the microsatellite-related miRNA-stem on miRNA family classification by pairwise alignments. The consensus sequence of MSHORT1 and RSHORT1 was included in the analysis to represent the C2MC miRNA genes. With an exception from MSHORT1/RSHORT1 and sequences of miRNA-1277, no sequence similarity was detected any other miRNA precursor sequence when the microsatellite content was masked (Additional File 2 Table S4B). Therefore, it is likely that miRNA genes in MSHORT1 and RSHORT1 have originated independently from all other miRNAs that are classified within miRNA gene families 466 and 467.

### 5. Secondary structure predicts a more stable hairpin in MSHORT1 than in RSHORT1

**Figure 4:**
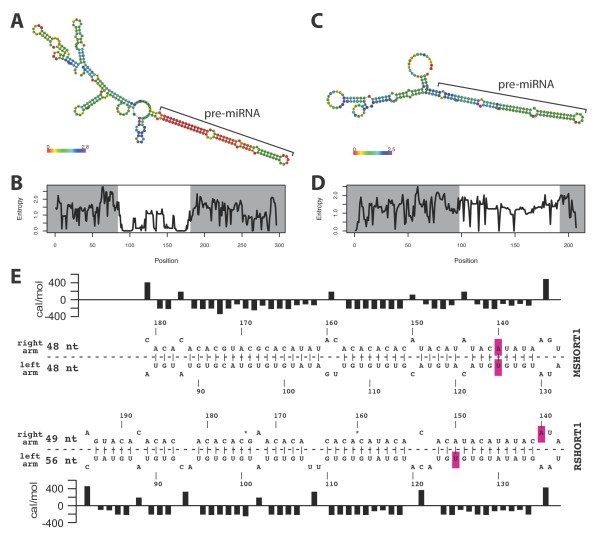
**Secondary structure prediction of RNA encoded by MSHORT1 and RSHORT1**. **A) **The model of the secondary structure of MSHORT1 is based on minimal free energy. The colors represent the positional entropy from low (red) to high (blue). The hairpin that forms pre-miRNAs of some MSHORT1 copies is indicated. **B) **Positional entropy is shown for the corresponding positions of the secondary structure. The pre-miRNA corresponding region is highlighted in white. **C) **&**D) **Same representation for the secondary structure of RSHORT1 (rats). Please note the region that potentially forms a pre-miRNA in MSHORT1 is more stable as compared to the corresponding region in RSHORT1. **E) **Detailed view of the predicted miRNA containing hairpin structures of MSHORT1 and RSHORT1. The two arms of the RSHORT1 hairpin contain a different number of nucleotides (left arm vs. right arm), while the number of nucleotides is equal in the two arms of the MSHORT1 hairpin. Energy rich, asymmetric bulges in the hairpin-stem of RSHORT1 result from an imbalance in hairpin arm length. In MSHORT1, intramolecular base pairing occurred between nt 125 and nt 140 (pink), while in RSHORT1 nt 125 pairs to nt 150. Sequence coordinates correspond to the pairwise alignment of the two sequences. The asterisk marks sites of deletion of two nucleotides in RSHORT1 compared to MSHORT1 (adapted from [32]).

The next point we addressed was the paradox that, although intron 10 of the rat and mouse genes contain mainly copies of SHORT1 including self-complementary microsatellites, the mouse has 65 miRNA-producing genes in this region, while the rat only has one gene that expresses Mir-466d. A possible explanation could be the stability of the secondary structures, which are considered relevant for pre-miRNA processing by Drosha and Dicer [31,32]. To address this point, we predicted the secondary mRNA structures from the rat and mouse SHORT1 consensus sequence (Figure 4A). The analysis was done using three structure prediction methods (minimal free energy structure, equilibrium base-pairing probabilities and centroid structure (Additional File 1 Figure S5)). For mouse and rat, a 41 nt double stranded hairpin was found that coincided with the predicted pre-miRNA sequence in the mouse. A major difference, however, was found in the entropy of the hairpin region, which was much lower as compared to immediate surroundings in the mouse than in the rat (Figure 4B). When we repeated the analysis without the extra 0.1 Kb at the 3' end of MSHORT1, a similar result was obtained as compared to full length MSHORT1 (data not shown). Therefore, we found a comparable overall stem-loop structure, but more pronounced differences in entropy in the region demarcating the 82-83 nt hairpin structure in the mouse MSHORT1. A detailed view on the RSHORT1 hairpin showed that next to point mutations, two deletions of two nucleotides caused a shift in the hairpin structure. For example, in RSHORT1 nt 125 does not pair to nt 140, as seen in MSHORT1, but to nt 150 (Figure 4C). Consequently, the RSHORT1 hairpin does not resemble the pre-miRNA structures of this miRNA cluster as closely as MSHORT1 does. Our results support the idea that both low entropy and structural resemblance is relevant for the pre-miRNA processing in the mouse.

## Discussion

In this study, we analyzed the repeat elements of C2MC, a mouse specific miRNA cluster in a large intron of a protein-encoding gene. The encoded protein SFMBT2 (Scm-like with four mbt domains 2) belongs to the polycomb protein family which is implicated in embryonic development [33]. Interestingly, the Sfmbt2-gene is imprinted and primarily expressed from the paternal allele in early embryos [34]. This makes another example of a large cluster of non-coding small RNA genes in regions of imprinted DNA [35,36]. In the analysis we performed in this paper, evidence was found for the idea that B1 transposons together with two self-complementary microsatellites, have generated a miRNA-containing cassette that has duplicated many times during a relatively short time of evolution. This idea fits well into an emerging concept that retroposon-driven local expansion of miRNA clusters can contribute to the miRNA repertoire of mammals [37,38]. In our analysis, the smallest repeat unit bearing one miRNA gene (MSHORT1) is composed of a 3' fragment of a B1F3 element, two microsatellites and additional sequence (Figure 2). The two microsatellites overlap with the miRNA gene of MSHORT1 and could have created a stable hairpin due to base pair complementarity. Perfect hairpins were proposed to be a source of de-novo generated plant miRNAs [10].

One can argue about the point whether or not the encoded small non-coding RNAs in C2MC are "real" miRNA's. In general, miRNA's are defined by a combination of five criteria for both expression and biogenesis (for review see [39]). Previous work has indicated that the miRNAs of C2MC fulfill three of these criteria [40-42]: (i) detection of a distinct expressed mature ~22 nt RNA transcript; (ii) the identification of its precise genomic match; (iii) the dependence on Dicer for miRNA production [39]. What is lacking for the C2MC mRNA's is phylogenetic conservation of the ~22 nt miRNA sequence and its predicted fold-back precursor [39]. Phylogenetic conservation may be exist, however as suggested by other miRNAs that are classified together with C2MC miRNA's into families 466 and 467) [43]. But one should be cautious about this as, we found that this family classification does largely depend on the microsatellite related origin of these miRNAs. While miRNAs of C2MC do contain a combination of (TG)n and (CA)n microsatellite, the other species' miRNAs grouped in these miRNA families generally involve a (TA)n microsatellite (Additional File 2 Table S4). Indeed, when we analyzed a 300 nt sequence with the microsatellite content masked, the miRNA genes of C2MC showed no sequence similarity to other miRNAs the 466 and 467 families. Therefore, the miRNAs of C2MC might have a different origin, and should perhaps be grouped in a separate miRNA family. An interesting detail is that mouse miRNAs of families 466 and 467 that do not belong to C2MC could not be confirmed in an experimental miRNA evaluation approach [42]. The last criterion for miRNA evaluation describes structural requirements for a hairpin precursor that contains the ~22 nt miRNA sequence. In this criterion, the hairpin must be the folding alternative with the lowest free energy and should not contain large internal loops or bulges, particularly not asymmetric bulges [39]. We have arguments that the small RNA's from C2MC are different from piRNAs (Piwi protein-associated small RNA's). First, piRNA gene clusters are present in intergenetic regions [44], while the small RNA's in C2MC are encoded in an intron of a protein-encoding gene. Second, the mature small RNAs of C2MC have a length of 22 nt, which is characteristic of miRNAs, and smaller as the length of piRNAs (24 nt to 31 nt) [45,46].

Differences between MSHORT1 and RSHORT1 should explain why the mouse miRNA cluster C2MC contains more miRNAs than its rat counterpart. In comparison to MSHORT1, the right arm of the RSHORT1 hairpin shifted laterally by 10 nt and gained 3 asymmetrical bulges, while MSHORT1 had none (Figure 4E). An energy profile of primary miRNA transcripts is described for humans. There, the region of the lower hairpin stem towards the basal segments and the upper stem close to the Drosha cleavage site were identified as thermodynamic most stable [31]. This energy profile is accentuated by the entropy profiles of MSHORT1, but it is less consistent with the profile of RSHORT1 (Figure 4). Further, sequences flanking the hairpins were described as essential for efficient in vitro processing [4,47]. However, RSHORT1 lacks this sequence on its 5 prime side (Additional File 1 Figure S3C). Together these differences might explain the low number of genes producing mature miRNAs in the rat cluster.

**Figure 5:**
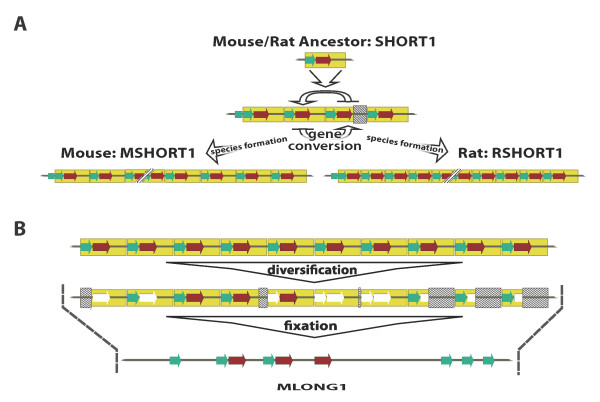
**Dual growth phase model of miRNA gene cluster in intron 10 of the Sfmbt2 gene**. **A) **During the first extension phase of the cluster in the last common ancestor of mouse and rat a 0.3 kb SHORT1 (yellow box) was copied by gene conversion. Ancestral SHORT1 contains the self-complementary microsatellite which may (mice) or may not (rats) evolve to miRNA genes (red arrow); this sequence is preceded by the 3' half of a B1F3-SINE (green arrow). A B1F3 full-length copy marks the 3' borders of both examined clusters. The initial unit diverged into MSHORT1 (0.3 kb) and RSHORT1 (0.2 kb), which are both detected in rat, while only MSHORT1 is found in mouse. **B) **Diversification of MSHORT1 in mouse caused a fixation of ten adjacent elements in the head-to-tail arrangement to create one 2.5 kb MLONG1 element (gray arrow), which was further amplified. Fixation involved sequence loss (dashed area) and mutation based element alienation beyond recognition of B1F3-elements and microsatellites (white arrow).

The results of BLAST analysis of MSHORT1 suggests a model for the evolution of C2MC (Figure 5). SHORT1 copies accumulated via a recombination-based mechanism in the common rodent ancestor of today's mice and rats (Figure 5A). These ancestral SHORT1 units may have been more similar to MSHORT1 than to RSHORT1. In fact, some repeat units in the cluster of modern rats resemble MSHORT1 instead of RSHORT1, while all repeat units in modern mice only resemble MSHORT1 (Additional File 2 Table S2 and S3). Thus, a truncation event resulting in the ~200nt RSHORT1 occurred in the rat after the speciation of mice and rats. We speculate that single nucleotide mutations and small deletion events in 10 neighboring MSHORT1 units, caused the formation of one larger unit MLONG1 (Figure 5B). MLONG1 was further multiplied in the C2MC center. This model is similar to what we proposed before in primates (27). In the mouse the individual miRNAs of C2MC have been duplicated together with a B1F3 retrotransposon.

What could be the biological implication of the growth of the C2MC miRNA gene cluster? One possibility is that such growth contributes to the well known expansion of a miRNA gene repertoire in mammals [14]. In order to regulate a great diversity of mRNA targets, a repertoire of "seeds" is needed. This requires a large set of miRNA genes. Local duplication events of miRNA genes in clusters are, therefore, to some extent analogous to a repertoire of variable immunoglobulin, T-cell receptor and Trypanosome variable surface glycoprotein genes [48-50]. A problem with this explanation is that the growth of the C2MC cluster has not yet generated new "seeds'" to regulate novel mRNA targets: the miRNAs from C2MC share the "seed" region within a group of five distinct miRNA clusters, present in mouse, human and zebra fish [51]. On the other hand, the miRNA genes may be evolutionary young and since they overlap with microsatellites, rapid evolution of the "seed" sequences may still occur. Another possibility of gene duplication is a dosage effect of miRNA's with the same seed. This is particularly interesting for imprinted genes such as Sfmbt2. More research is needed to evaluate the impact of miRNA concentration generated by the miRNA genes in C2MC and to compare this action with that other amplified miRNA clusters, e.g. on chromosome 19 in primates.

## Conclusion

In summary, we analyzed C2MC, a large mouse miRNA cluster that expanded during the time since mice and rats diverged from each other. Our data indicate that a B1F3 retrotransposon has contributed to the generation of a tandemly repeated unit MSHORT1, which grouped into the 2.5 KB MLONG1 element. The latter led to rapid growth in the center of the cluster by further duplication events. Our study indicates that C2MC is a new example of the phenomenon in which lineage-specific SINE elements contribute to growth of the miRNA gene repertoire in a particular species.

## Methods

### Selection of pre-miRNAs with 3' and 5' flanking sequence

MicroRNA precursor coordinates were selected from the Sanger miRNA Registry database (version 16.0). All pre-miRNA sequences of miRNA family 466 and 467 were extracted with adjacent 100nt at the 5' and 3' borders, in those cases where the pre-miRNAs were not in present within the mouse and rat miRNA cluster. The sequences were selected from the mouse (release mm9), rat genome (release rn4), chicken (release galGal4), human (release hg19), pig (release susScr2) and orangutan (release ponAbe2) from the UCSC genome browser http://genome.ucsc.edu/. The microsatellite content of these sequences was masked with RepeatMasker using the "mask only complex/simple repeats" option.

### Generation of multiple and pairwise alignments

Multiple alignments were calculated with ClustalX version 2.0.10 (ftp.embl-heidelberg.de) using the IUB DNA weight matrix with the following variables: gap opening 10, gap extension 0.2, delay divergent sequence 30%, DNA transition weight -0.5 and no negative matrix. The alignments were generated from the mouse MSHORT1 copies and rat MSHORT1 RSHORT1 copies (Additional File 1 Figures S3, S4). Multiple alignments were edited with the SEAVIEW program [52] and used in the phylogenetic analyses. Pairwise alignments were calculated with maln http://www.girinst.org for miRNA precursors with 100nt 5' and 3' sequence of miRNA family 466 and 467 that are not located in this miRNA cluster of mouse and rat.

### Generation of the consensus sequences MSHORT1 and RSHORT1

The selection of initial sequences was guided by the regular spaced miRNA genes (Figure 1C). The selection was limited to 4 Kb in order to minimize overlap with down or upstream positioned regular spaced set of 3 or 2 miRNA genes. Multiple alignment was calculated from these sequences with ClustalX version 2.0.10 (ftp.embl-heidelberg.de) using the IUB DNA weight matrix. A similarity graph was used to access the quality of the multiple alignment and to define a primary consensus sequence. "Similarity" is a method distributed with the VectorNTI suite from Invitrogen http://www.invitrogen.com/vectornti. This consensus sequence was used to identify MSHORT1 and RSHORT1. A series of Censor [53] analyses were applied to sharpen consensus boundaries of these 2 consensus sequences. The quality of Censor identified sequences was controlled in pairwise comparisons calculated with maln http://www.girinst.org, before including the sequences in the next analysis cycle. The B1F3 element was identified with Censor. The B1F3 element was probably an ancestral B1 element that gave birth to the ID_B1 family by fusion with a tRNA-like ID element (Kapitonov and Jurka, submitted to Repbase Reports as the B1F3 family consensus sequence). The mouse and rat Sfmbt2 intron 10 composition of MSHORT1, RSHORT1 and B1F3 elements is summarized in Additional File 2 Table S2 and S3. Censor was also used to analyze the entire genomes of Mouse, rat, human, chicken, *D. melanogaster*, *C. elegans *for MSHORT1 copies. Further, the sequence of MLONG1, which was indicated by the phylogenetic clustering of MSHORT1 copies, was verified with Censor (Figure 2A).

### Phylogenetic trees and graphical visualization of repeat elements and miRNAs features on selected sequences

Phylogenetic analyses were conducted in MEGA4 [54] using a Neighbor-Joining method with a pair-wise deletion model [1]. The bootstrap consensus tree inferred from 1000 replicates [2]. The percentage of replicate trees in which the associated taxa clustered together was calculated with the bootstrap test (1000 replicates) [2]. The evolutionary distances were computed using the Maximum Composite Likelihood method [3] and are in units of the number of base substitutions per site. All positions containing alignment gaps and missing data were eliminated only in pairwise sequence comparisons (pairwise deletion option). Secondary structure predictions were performed with the Vienna RNA web suite [32]. Repeat features were selected from the mouse (release mm9) and rat genome (release rn4; http://genome.ucsc.edu/). miRNA features were extracted from the Sanger miRNA Registry (version 15.0). Perl was used to generate customized Genbank formatted feature files, which were subsequently used to generate graphs visualizing exact positions and length of the features on each sequence. Graphical prints of the sequences were generated with the DNAPlotter [55] and Vector NTI 11.0 http://www.invitrogen.com/vectornti.

## Abbreviations

C2MC: (mouse chromosome 2 miRNA gene cluster); MSHORT1, MLONG1: (specific repeat elements of 0.3 and 2.5 Kb respectively in intron 10 of the mouse Sfmbt2 gene); SINE: (short interspersed nuclear elements).

## Competing interests

The authors declare that they have no competing interests.

## Authors' contributions

SL, VK and FCS conceived and designed the experiments, data analysis was performed by SL, VK and PJT and the manuscript was written by SL and FCS. All authors have read and approved the final manuscript.

## Supplementary Material

Additional File 1 **Lehnert_et_al_Supplement_Figures.pdf**. Click here for file

Additional File 2 **Lehnert_et_al_Supplement_Tabels.pdf**. Click here for file
