# Supplementary material for: Modeling the asymmetric evolution of a mouse and rat-specific microRNA gene cluster intron 10 of the Sfmbt2 gene
Source: BMC Genomics. 2011 May 23;12:257. doi: 10.1186/1471-2164-12-257 (PMC3212979; doi:10.1186/1471-2164-12-257)
Supplement: Additional File 1 — Lehnert_et_al_Supplement_Figures.pdf. [file 1471-2164-12-257-S1.PDF]

Modeling of the asymmetric evolution of one mouse and rat-specific microRNA gene cluster in the Sfmbt2 locus

Supplement Figures

Stefan Lehnert[1] (Stefan.Lehnert@med.kuleuven.be),

Vladimir Kapitonov[2] (vladimir@girinst.org),

Pushpike J. Thilakarathne[3] (Pushpike@med.kuleuven.be),

Frans C. Schuit[1] (Frans.Schuit@med.kuleuven.be)

[1] Gene Expression Unit, Department of Molecular Cell Biology, Katholieke Universiteit Leuven;

[2] Genetic Information Research Institute 1925 Landings Dr Mountain View, CA 94043, Unites States of America

[3] Interuniversity Institute for Biostatistics and statistical Bioinformatics, Katholieke Universiteit Leuven, Kapucijnenvoer 35, Blok D, bus 7001, B3000 Leuven, Belgium, and Universiteit Hasselt, Belgium;

Address correspondence and reprint requests to Frans C. Schuit, Herestraat 49, P.O. Box 901, B-3000 Leuven, Belgium. E-mail: frans.schuit@med.kuleuven.be

Keywords: microRNA, miRNA, simple repeat, SINE B1F3, evolution, gene conversion.

Lehnert et al Supplement Figure 1

A)

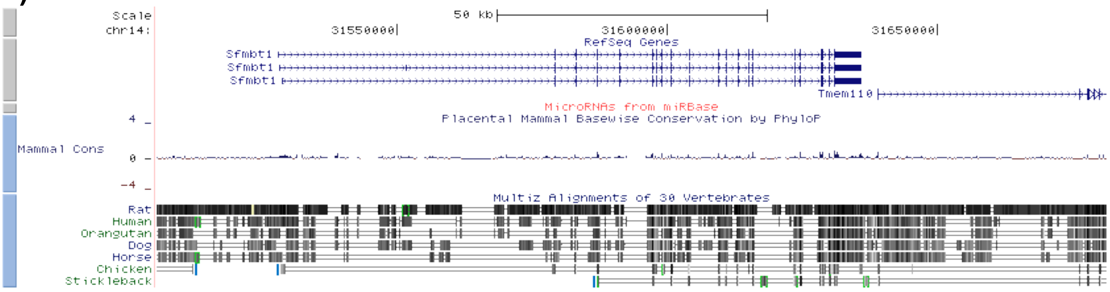

B)

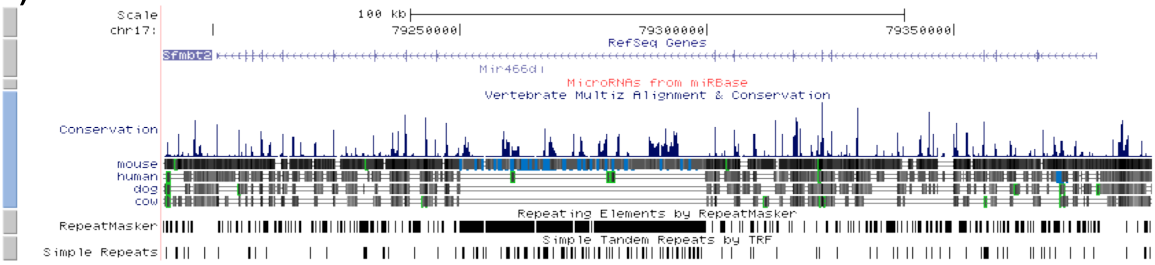

C)

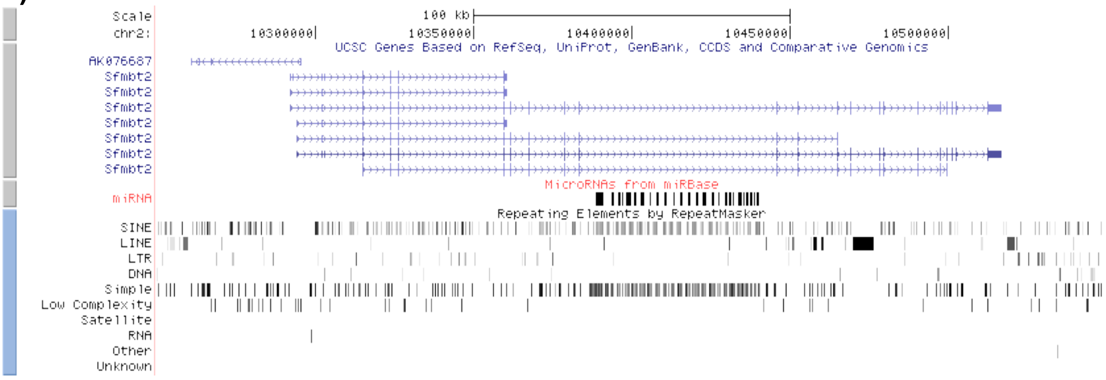

Supplement Figure 1: Mouse Sfmbt2 paralogous gene Sfmbt1, rat orthologous Sfmbt2 gene and Mouse Sfmbt2 repeat element contend

A) Overview of the paralogous mouse gene *Sfmbt1* is shown using the mouse UCSC genome browser (mouse chr14:31,505,697-31,681,201; Version mm9). No MiRNAs were found. **B)** The orthologous Rat *Sfmbt2* gene is associated with one miRNA gene in the intron 10 (rat chr17:79,190,001-79,390,000; version rn4). **C)** Repeat elements and miRNAs are clustered in intron 10 of the mouse Sfmbt2 gene (mouse chr2:10,250,001-10,550,000; Version mm9). Figures are shown using the UCSC genome browser (http://genome.ucsc.edu).

Lehnert et al Supplement Figure 2

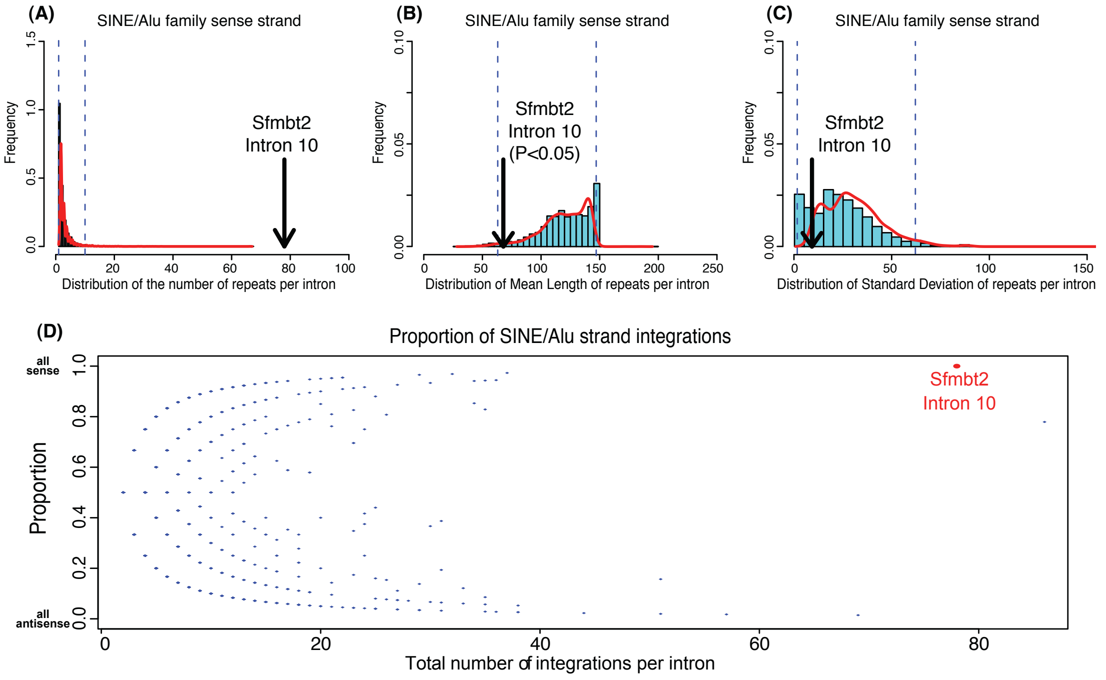

Supplement Figure 2: SINE Alu family elements in the Sfmbt2 intron 10 locus are more frequent, significant smaller and unequally distributed over the two DNA strands

A) The frequency distribution histogram of SINE Alu family sense repeats is shown per intron. All introns of all genes on chromosome 2 were selected (transcript variants removed) and compared to the number of the Sfmbt2 intron 10 SINE Alu family sense repeats. B1F3 repeats belong to the SINE Alu family. The superimposed red curved line indicates the kernel density estimate for the distribution. The observed number in the Sfmbt2 intron 10, indicated with an arrow is largest of all analyzed introns. A P-value is not reported, as the kernel density estimate does not follow an approximately symmetric distribution. Panel (**B**) and (**C**) are similar to (**A**), but mean length (**B**) and standard deviation (**C**) of SINE Alu family elements in the Sfmbt2 intron 10 are shown. The mean length was significantly smaller as compared to random nucleotide samples taken from the other introns of chromosome 2. **D**) SINE Alu family elements are unequally distributed in the Sfmbt2 intron 10. Analysis is based on the same random sampled windows, but SINE Alu family repeats are selected in both orientations (sense and antisense) from the introns in regard to the strand of transcription. For each window the proportion of integration is computed, with respect to the total integration of that window (blue rhombus; 0 equals 100% of integrations in antisense orientation, 1 equals 100% in sense orientation). Proportions of the individual windows are plotted in regard to their total integration number from left to right ascending. The red dot shows the proportion of the Sfmbt2 intron 10.

Lehnert et al Supplement Figure 3

A

```
MSHORT1
GAGTCAGGAGGATCAGGAGTTCAAATTCATGCTTAACTTCATGATGAGTTAGAGATCAGCCTGGGATTCA
TGTGATGCCCCCCATGTATGTGCATGTGTGTATAGTTGTGTGTGCATGTACATGTGTGTATATGAATATA
CATATACATACACACACACATATACACGCATGCACACACACACACACAGGAATGGCACTCATCGATCCAT
TTGAATGATATGTGACTTAGAGTTTTAGAGATTATGCAGTACTGGGCCAGTGAAAGGTCTCACTCACCAG
GTGAAAACGCTTGCCA
```

B

```
>RSHORT1
AACACTTGGGAGACAGAGTCAGGGGGATCAGGAGTTCAAACTCATGCTTCACTTCATGATGAGTTAGAAA
TCAGCCTGGGATTCATGTGATGCCCCTATGTATGTGCATGTGTGTATGTGTTTGTGTGTATGTACATGTG
TGTATATGAATATACATATACATACACACATACACACACACAAGCACACACACACACATGAATGGCA
```

C

```
top: MSHORT1  1 - 197

bot: RSHORT1  16 - 207

1        10        20        30        40        50        60        70
|2 4 6 8 | 2 4 6 8 | 2 4 6 8 | 2 4 6 8 | 2 4 6 8 | 2 4 6 8 | 2 4 6 8 |
GAGTCAGGAGGATCAGGAGTTCAAATTCATGCTTAACTTCATGATGAGTTAGAGATCAGCCTGGGATTCA
********|****************|******** ******************|****************
GAGTCAGGGGGATCAGGAGTTCAAACTCATGCTTCACTTCATGATGAGTTAGAAATCAGCCTGGGATTCA

71       80        90        100       110       120       130       140
|2 4 6 8 | 2 4 6 8 | 2 4 6 8 | 2 4 6 8 | 2 4 6 8 | 2 4 6 8 | 2 4 6 8 |
TGTGATGCCCCCCATGTATGTGCATGTGTGTATAGTTGTGTGTGCATGTACATGTGTGTATATGAATATA
******* ****|********************|  * ******|*************************
TGTGATG-CCCCTATGTATGTGCATGTGTGTATGTGTTTGTGTGTATGTACATGTGTGTATATGAATATA

141      150       160       170       180       190
|2 4 6 8 | 2 4 6 8 | 2 4 6 8 | 2 4 6 8 | 2 4 6 8 | 2 4 6
CATATACATACACACACACATATACACGCATGCACACACACACACACAGGAATGGCA
****************|**  *|****|** *  ************** ********
CATATACATACACACATAC--ACACACACAAG--CACACACACACACATGAATGGCA

MSHORT1 - RSHORT1: Identity = 0.91
```

Supplement Figure 3. Consensus sequences of mouse MSHORT1 and rat RSHORT1 and their pair wise alignment

A) A 296 nt consensus of MSHORT1 was derived from multiple alignments of 193 copies of MSHORT1 tandemly repeated in intron 10 of the mouse Sfmbt2 gene. **B)** A 207 nt consensus of RSHORT1 was derived from multiple alignments of 236 copies of RSHORT1 tandemly repeated in intron 10 of the rat Sfmbt2 gene. **C)** Pair wise alignment of the two consensus sequences, matches are marked by *, transitions are marked by |.

A

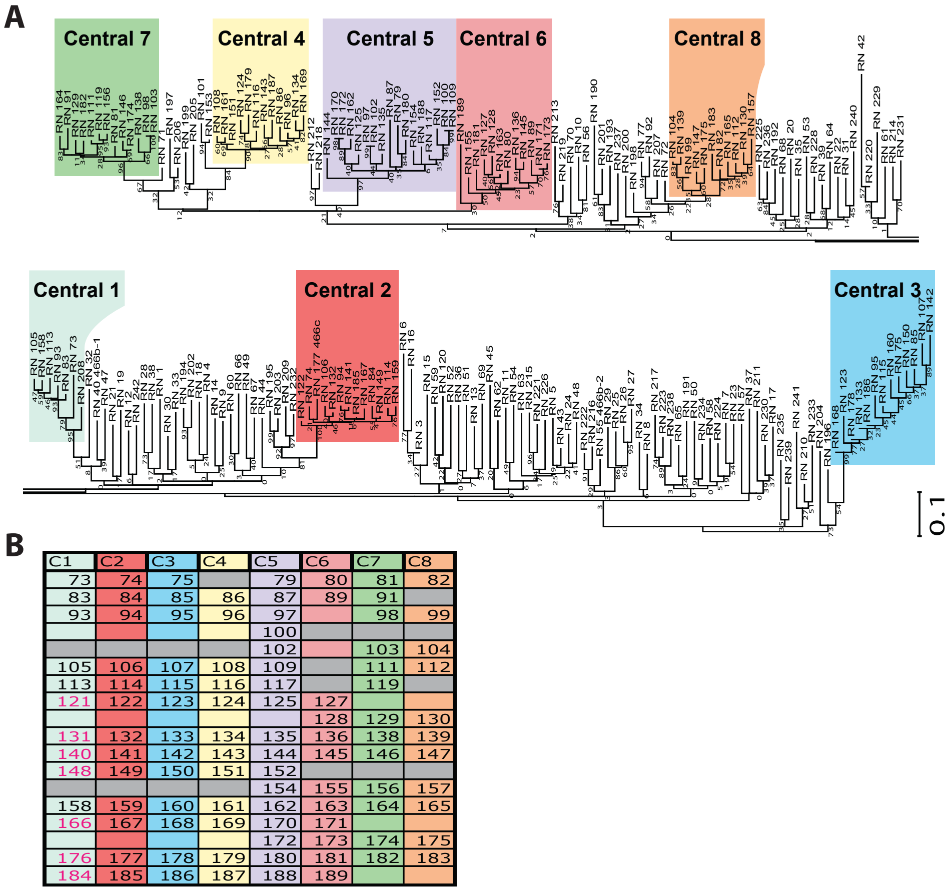

B

C1	C2	C3	C4	C5	C6	C7	C8
73	74	75		79	80	81	82
83	84	85	86	87	89	91	
93	94	95	96	97		98	99
				100			
				102		103	104
105	106	107	108	109		111	112
113	114	115	116	117		119	
121	122	123	124	125	127		
					128	129	130
131	132	133	134	135	136	138	139
140	141	142	143	144	145	146	147
148	149	150	151	152			
				154	155	156	157
158	159	160	161	162	163	164	165
166	167	168	169	170	171		
				172	173	174	175
176	177	178	179	180	181	182	183
184	185	186	187	188	189		

Supplement Figure 4. Evolutionary relationships of 233 rat RSHORT1 copies

A) The evolutionary history was inferred using the Neighbor-Joining method [1]. The bootstrap consensus tree inferred from 1000 replicates [2] is taken to represent the evolutionary history of the RSHORT1 copies analyzed [2]. RSHORT1 copies are numbered from left to right for their location in the mouse miRNA cluster. The copies belonging to the central region of the cluster are color-coded. Branches corresponding to partitions reproduced in less than 50% bootstrap replicates are collapsed. The percentage of replicate trees in which the associated taxa clustered together in the bootstrap test (1000 replicates) is shown next to the branches [2]. The tree is drawn to scale, with branch lengths in the same units as those of the evolutionary distances used to infer the phylogenetic tree. The evolutionary distances were computed using the Maximum Composite Likelihood method [3] and are in units of the number of base substitutions per site. All positions containing alignment gaps and missing data were eliminated only in pair wise sequence comparisons (Pair wise deletion option). There were a total of 416 positions in the final data set. Phylogenetic analyses were conducted in MEGA4 [4]. **B)** The Sequential order of RSHORT1 copies is highlighted. The RSHORT1 units that belong to the same sub-cluster are listed in columns. Rows show the arrangement of RSHORT1 units in the corresponding long copies. Gray boxed show RSHORT1 copies that did not classify to either one of the rat subgroups C1 to C8; not numbered fields represent non-existing copies of RSHORT1; red numbered

units were too short to be included in the phylogenetic analysis, but matched to C1 with their remaining sequence.

1. Saitou N & Nei M (1987) The neighbor-joining method: A new method for reconstructing phylogenetic trees. Molecular Biology and Evolution 4:406-425.

2. Felsenstein J (1985) Confidence limits on phylogenies: An approach using the bootstrap. Evolution 39:783-791.

3. Tamura K, Nei M & Kumar S (2004) Prospects for inferring very large phylogenies by using the neighbor-joining method. Proceedings of the National Academy of Sciences (USA) 101:11030-11035.

4. Tamura K, Dudley J, Nei M & Kumar S (2007) MEGA4: Molecular Evolutionary Genetics Analysis (MEGA) software version 4.0. Molecular Biology and Evolution 24:1596-1599.

Lehnert et al Supplement Figure 5

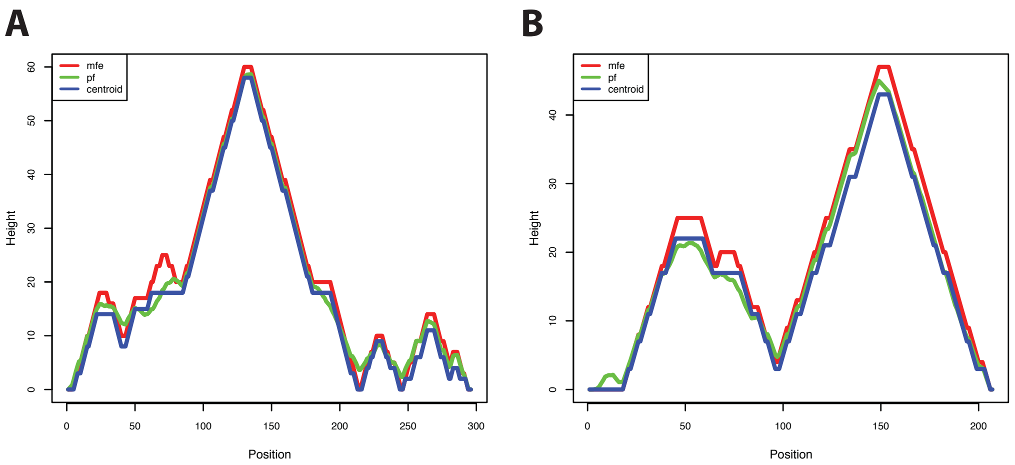

Supplement Figure 5: Secondary structure prediction of MSHORT1 and RSHORT1

Mountain plots of MSHORT1 **(A)** and RSHORT1 **(B)** calculated from three structure prediction methods: minimal free energy structure (mfe - red); equilibrium base-pairing probabilities (pf - green); centroid structure (centroid - blue), the structure with minimal base-pair distance to all structures in the thermodynamic ensemble. Single stranded loops correspond to plateaus (hairpin loops are peaks) and helices to slopes.
