# Supplementary material for: Modeling the asymmetric evolution of a mouse and rat-specific microRNA gene cluster intron 10 of the Sfmbt2 gene
Source: BMC Genomics. 2011 May 23;12:257. doi: 10.1186/1471-2164-12-257 (PMC3212979; doi:10.1186/1471-2164-12-257)
Supplement: Additional File 2 — Lehnert_et_al_Supplement_Tabels.pdf. [file 1471-2164-12-257-S2.PDF]

**Modeling of the asymmetric evolution of one mouse and rat-specific microRNA gene cluster in the Sfmbt2 locus**

# Supplement Tables

Stefan Lehnert[1] (Stefan.Lehnert@med.kuleuven.be),

Vladimir Kapitonov[2] (vladimir@girinst.org),

Pushpike J. Thilakarathne[3] (Pushpike@med.kuleuven.be),

Frans C. Schuit[1] (Frans.Schuit@med.kuleuven.be)

[1] Gene Expression Unit, Department of Molecular Cell Biology, Katholieke Universiteit Leuven;

[2] Genetic Information Research Institute 1925 Landings Dr Mountain View, CA 94043, Unites States of America

[3] Interuniversity Institute for Biostatistics and statistical Bioinformatics, Katholieke Universiteit Leuven, Kapucijnenvoer 35, Blok D, bus 7001, B3000 Leuven, Belgium, and Universiteit Hasselt, Belgium;

Address correspondence and reprint requests to Frans C. Schuit, Herestraat 49, P.O. Box 901, B-3000 Leuven, Belgium. E-mail: frans.schuit@med.kuleuven.be

Keywords: microRNA, miRNA, simple repeat, SINE B1F3, evolution, gene conversion.

```
mouse   chr2 10383102 10445570
rat     chr17 79301645 79245165
kangaroo_rat GeneScaffold_6552 152744 143027
rabbit   AAGW02026857 41410 53848
gpig     scaffold_16  16013390 16026898

Genome assemblies
=================
Guinea pig (Cavia porcellus):
ftp://ftp.ensembl.org/pub/release-
58/fasta/cavia_porcellus/dna/Cavia_porcellus.cavPor3.58.dna.nonchromosomal.fa.gz

Kangaroo rat (Dipodomys_ordii):
ftp://ftp.ensembl.org/pub/release-
55/fasta/dipodomys_ordii/dna/Dipodomys_ordii.dipOrd1.55.dna.nonchromosomal.fa.gz

Ground squirrel (Spermophilus_tridecemlineatus)
ftp://ftp.ensembl.org/pub/release-
58/fasta/spermophilus_tridecemlineatus/dna/Spermophilus_tridecemlineatus.SQUIRREL.58.d
na.nonchromosomal.fa.gz


Ground squirrel:
scaffold_5659   10089 10196   Sfmbt2           289 324     d   0.7778
scaffold_5659   12816 12968   Sfmbt2           323 373     d   0.8039

GeneScaffold_5788   61782 61913         Sfmbt2     853 896     c   0.7500
GeneScaffold_5788   62856 63197         Sfmbt2     746 858     c   0.4435
GeneScaffold_5788   64981 65175         Sfmbt2     600 664     c   0.7846
GeneScaffold_5788   74912 75028         Sfmbt2     565 603     c   0.7692
GeneScaffold_5788   97330 97524         Sfmbt2     498 567     c   0.6970
GeneScaffold_5788  103073 103180        Sfmbt2     442 477     c   0.8056
GeneScaffold_5788  113670 113789        Sfmbt2     396 435     c   0.7750
GeneScaffold_5788  119783 119866        Sfmbt2     373 400     c   0.7857
GeneScaffold_5788  166031 166120        Sfmbt2     145 174     c   0.8333
GeneScaffold_5788  234259 234486        Sfmbt2      62 138     c   0.7143
```

***Supplement Table 1. Assembly of mouse Sfmbt2 intron 10 in rat, kangaroo rat, rabbit, guinea pig and ground squirrel.***

Intron 10 in ground squirrel was assembled from 2 contigs.

| Nr | | L1 | L2 | | N1 | N2 | D | Identity | Score |
|---|---|---|---|---|---|---|---|---|---|
| | intron10_MM | 3153 | 3269 | B1F3 | 4 | 120 | d | 0.7350 | 509 |
| 1 | intron10_MM | 3195 | 3574 | MSHORT1 | 1 | 296 | d | 0.8669 | 1442 |
| 2 | intron10_MM | 3580 | 3817 | MSHORT1 | 12 | 259 | d | 0.8683 | 1460 |
| 3 | intron10_MM | 3818 | 3910 | MSHORT1 | 82 | 196 | d | 0.8646 | 446 |
| 4 | intron10_MM | 3952 | 4215 | MSHORT1 | 3 | 296 | d | 0.8390 | 1305 |
| 5 | intron10_MM | 4216 | 4523 | MSHORT1 | 1 | 296 | d | 0.8356 | 1520 |
| 6 | intron10_MM | 4525 | 4809 | MSHORT1 | 2 | 296 | d | 0.8357 | 1592 |
| 7 | intron10_MM | 4810 | 5094 | MSHORT1 | 1 | 296 | d | 0.8211 | 1459 |
| 8 | intron10_MM | 5095 | 5395 | MSHORT1 | 1 | 296 | d | 0.8754 | 1863 |
| 9 | intron10_MM | 5396 | 5670 | MSHORT1 | 1 | 296 | d | 0.8993 | 1773 |
| 10 | intron10_MM | 5671 | 5963 | MSHORT1 | 1 | 296 | d | 0.8946 | 1998 |
| 11 | intron10_MM | 5971 | 6232 | MSHORT1 | 9 | 296 | d | 0.8453 | 1370 |
| 12 | intron10_MM | 6236 | 6507 | MSHORT1 | 4 | 292 | d | 0.8667 | 1597 |
| 13 | intron10_MM | 6513 | 6811 | MSHORT1 | 2 | 294 | d | 0.8237 | 1478 |
| 14 | intron10_MM | 6813 | 7114 | MSHORT1 | 1 | 296 | d | 0.8836 | 1795 |
| 15 | intron10_MM | 7115 | 7411 | MSHORT1 | 1 | 296 | d | 0.8938 | 1880 |
| 16 | intron10_MM | 7412 | 7714 | MSHORT1 | 1 | 293 | d | 0.8644 | 1784 |
| 17 | intron10_MM | 7719 | 8013 | MSHORT1 | 2 | 296 | d | 0.8407 | 1668 |
| 18 | intron10_MM | 8400 | 8646 | MSHORT1 | 15 | 296 | d | 0.8367 | 1229 |
| 19 | intron10_MM | 8647 | 8943 | MSHORT1 | 1 | 296 | d | 0.8490 | 1674 |
| 20 | intron10_MM | 8944 | 9257 | MSHORT1 | 1 | 296 | d | 0.7850 | 1168 |
| 21 | intron10_MM | 9258 | 9523 | MSHORT1 | 1 | 296 | d | 0.8074 | 1131 |
| 22 | intron10_MM | 9524 | 9792 | MSHORT1 | 1 | 296 | d | 0.8309 | 1252 |
| 23 | intron10_MM | 9793 | 10078 | MSHORT1 | 1 | 293 | d | 0.8448 | 1619 |
| 24 | intron10_MM | 10082 | 10382 | MSHORT1 | 1 | 286 | d | 0.8997 | 1859 |
| 25 | intron10_MM | 10393 | 10686 | MSHORT1 | 1 | 296 | d | 0.8767 | 1807 |
| 26 | intron10_MM | 10687 | 10978 | MSHORT1 | 1 | 296 | d | 0.8464 | 1668 |
| 27 | intron10_MM | 10979 | 11258 | MSHORT1 | 1 | 293 | d | 0.7817 | 1194 |
| 28 | intron10_MM | 11262 | 11540 | MSHORT1 | 1 | 296 | d | 0.8432 | 1496 |
| 29 | intron10_MM | 11541 | 11808 | MSHORT1 | 1 | 296 | d | 0.8339 | 1287 |
| 30 | intron10_MM | 11809 | 11971 | MSHORT1 | 1 | 167 | d | 0.8193 | 818 |
| 31 | intron10_MM | 12025 | 12365 | MSHORT1 | 6 | 296 | d | 0.7811 | 1150 |
| 32 | intron10_MM | 12366 | 12650 | MSHORT1 | 1 | 293 | d | 0.8179 | 1412 |
| 33 | intron10_MM | 12654 | 12885 | MSHORT1 | 1 | 233 | d | 0.8340 | 1249 |
| 34 | intron10_MM | 12900 | 13178 | MSHORT1 | 1 | 296 | d | 0.8582 | 1546 |
| 35 | intron10_MM | 13180 | 13450 | MSHORT1 | 2 | 283 | d | 0.8388 | 1409 |
| 36 | intron10_MM | 13462 | 13755 | MSHORT1 | 10 | 296 | d | 0.7958 | 1243 |
| 37 | intron10_MM | 13756 | 13919 | MSHORT1 | 1 | 166 | d | 0.8061 | 786 |
| 38 | intron10_MM | 13969 | 14128 | MSHORT1 | 1 | 162 | d | 0.8012 | 765 |
| 39 | intron10_MM | 14185 | 14471 | MSHORT1 | 4 | 296 | d | 0.7762 | 1139 |
| 40 | intron10_MM | 14472 | 14779 | MSHORT1 | 1 | 296 | d | 0.8249 | 1528 |
| 41 | intron10_MM | 14782 | 15062 | MSHORT1 | 3 | 293 | d | 0.8097 | 1350 |
| 42 | intron10_MM | 15066 | 15297 | MSHORT1 | 1 | 233 | d | 0.8298 | 1244 |
| 43 | intron10_MM | 15312 | 15590 | MSHORT1 | 1 | 296 | d | 0.8582 | 1555 |
| 44 | intron10_MM | 15592 | 15860 | MSHORT1 | 2 | 283 | d | 0.8212 | 1265 |
| 45 | intron10_MM | 15872 | 16165 | MSHORT1 | 10 | 296 | d | 0.7924 | 1230 |
| 46 | intron10_MM | 16166 | 16329 | MSHORT1 | 1 | 166 | d | 0.8242 | 840 |
| 47 | intron10_MM | 16381 | 16538 | MSHORT1 | 3 | 162 | d | 0.8553 | 906 |
| 48 | intron10_MM | 16595 | 16927 | MSHORT1 | 4 | 296 | d | 0.7677 | 1057 |
| 49 | intron10_MM | 16928 | 17236 | MSHORT1 | 1 | 296 | d | 0.8148 | 1444 |
| 50 | intron10_MM | 17239 | 17520 | MSHORT1 | 3 | 293 | d | 0.8166 | 1401 |
| 51 | intron10_MM | 17524 | 17755 | MSHORT1 | 1 | 233 | d | 0.8298 | 1235 |
| 52 | intron10_MM | 17770 | 18048 | MSHORT1 | 1 | 296 | d | 0.8546 | 1530 |
| 53 | intron10_MM | 18050 | 18316 | MSHORT1 | 2 | 283 | d | 0.8139 | 1261 |
| 54 | intron10_MM | 18328 | 18620 | MSHORT1 | 10 | 296 | d | 0.7889 | 1201 |
| 55 | intron10_MM | 18621 | 18784 | MSHORT1 | 1 | 166 | d | 0.8121 | 812 |
| 56 | intron10_MM | 18836 | 19005 | MSHORT1 | 3 | 172 | d | 0.8412 | 912 |
| 57 | intron10_MM | 19050 | 19372 | MSHORT1 | 4 | 296 | d | 0.7559 | 1101 |
| 58 | intron10_MM | 19373 | 19681 | MSHORT1 | 1 | 296 | d | 0.8289 | 1541 |
| 59 | intron10_MM | 19684 | 19966 | MSHORT1 | 3 | 293 | d | 0.8131 | 1370 |
| 60 | intron10_MM | 19970 | 20201 | MSHORT1 | 1 | 233 | d | 0.8468 | 1296 |
| 61 | intron10_MM | 20216 | 20494 | MSHORT1 | 1 | 296 | d | 0.8440 | 1484 |
| 62 | intron10_MM | 20496 | 20762 | MSHORT1 | 2 | 283 | d | 0.8029 | 1206 |
| 63 | intron10_MM | 20774 | 21056 | MSHORT1 | 10 | 285 | d | 0.7950 | 1212 |
| 64 | intron10_MM | 21068 | 21231 | MSHORT1 | 1 | 166 | d | 0.8121 | 802 |
| 65 | intron10_MM | 21283 | 21439 | MSHORT1 | 3 | 162 | d | 0.8491 | 892 |
| 66 | intron10_MM | 21496 | 21808 | MSHORT1 | 4 | 296 | d | 0.7612 | 1074 |
| 67 | intron10_MM | 21809 | 22116 | MSHORT1 | 1 | 296 | d | 0.8289 | 1551 |
| 68 | intron10_MM | 22119 | 22401 | MSHORT1 | 3 | 293 | d | 0.8131 | 1391 |
| 69 | intron10_MM | 22405 | 22636 | MSHORT1 | 1 | 233 | d | 0.8340 | 1250 |
| 70 | intron10_MM | 22651 | 22929 | MSHORT1 | 1 | 296 | d | 0.8546 | 1530 |
| 71 | intron10_MM | 22931 | 23201 | MSHORT1 | 2 | 283 | d | 0.8007 | 1251 |
| 72 | intron10_MM | 23213 | 23506 | MSHORT1 | 10 | 296 | d | 0.7889 | 1215 |
| 73 | intron10_MM | 23507 | 23669 | MSHORT1 | 1 | 166 | d | 0.8242 | 849 |

| | | | | | | | | | | |
|---|---|---|---|---|---|---|---|---|---|---|
| 74 | intron10_MM | 23721 | 23878 | MSHORT1 | 3 | 162 | d | 0.8679 | 945 |
| 75 | intron10_MM | 23935 | 24265 | MSHORT1 | 4 | 296 | d | 0.7492 | 1030 |
| 76 | intron10_MM | 24266 | 24573 | MSHORT1 | 1 | 296 | d | 0.8148 | 1475 |
| 77 | intron10_MM | 24576 | 24858 | MSHORT1 | 3 | 293 | d | 0.8166 | 1396 |
| 78 | intron10_MM | 24862 | 25093 | MSHORT1 | 1 | 233 | d | 0.8298 | 1236 |
| 79 | intron10_MM | 25108 | 25386 | MSHORT1 | 1 | 296 | d | 0.8617 | 1569 |
| 80 | intron10_MM | 25388 | 25657 | MSHORT1 | 2 | 283 | d | 0.8116 | 1294 |
| 81 | intron10_MM | 25669 | 25962 | MSHORT1 | 10 | 296 | d | 0.8028 | 1266 |
| 82 | intron10_MM | 25963 | 26126 | MSHORT1 | 1 | 166 | d | 0.8242 | 850 |
| 83 | intron10_MM | 26181 | 26332 | MSHORT1 | 9 | 162 | d | 0.8366 | 820 |
| 84 | intron10_MM | 26389 | 26732 | MSHORT1 | 4 | 296 | d | 0.7700 | 1072 |
| 85 | intron10_MM | 26733 | 27037 | MSHORT1 | 1 | 296 | d | 0.8243 | 1463 |
| 86 | intron10_MM | 27040 | 27320 | MSHORT1 | 3 | 293 | d | 0.8166 | 1368 |
| 87 | intron10_MM | 27324 | 27555 | MSHORT1 | 1 | 233 | d | 0.8419 | 1253 |
| 88 | intron10_MM | 27570 | 27850 | MSHORT1 | 1 | 296 | d | 0.8622 | 1553 |
| 89 | intron10_MM | 27852 | 28121 | MSHORT1 | 2 | 283 | d | 0.8116 | 1281 |
| 90 | intron10_MM | 28133 | 28426 | MSHORT1 | 10 | 296 | d | 0.7924 | 1234 |
| 91 | intron10_MM | 28427 | 28590 | MSHORT1 | 1 | 166 | d | 0.8242 | 842 |
| 92 | intron10_MM | 28638 | 28795 | MSHORT1 | 3 | 162 | d | 0.8679 | 934 |
| 93 | intron10_MM | 28852 | 29178 | MSHORT1 | 4 | 296 | d | 0.7500 | 1039 |
| 94 | intron10_MM | 29179 | 29486 | MSHORT1 | 1 | 296 | d | 0.8289 | 1551 |
| 95 | intron10_MM | 29489 | 29771 | MSHORT1 | 3 | 293 | d | 0.8131 | 1391 |
| 96 | intron10_MM | 29775 | 30006 | MSHORT1 | 1 | 233 | d | 0.8340 | 1250 |
| 97 | intron10_MM | 30021 | 30299 | MSHORT1 | 1 | 296 | d | 0.8546 | 1530 |
| 98 | intron10_MM | 30301 | 30571 | MSHORT1 | 2 | 283 | d | 0.8007 | 1251 |
| 99 | intron10_MM | 30583 | 30876 | MSHORT1 | 10 | 296 | d | 0.7889 | 1215 |
| 100 | intron10_MM | 30877 | 31039 | MSHORT1 | 1 | 166 | d | 0.8242 | 849 |
| 101 | intron10_MM | 31091 | 31248 | MSHORT1 | 3 | 162 | d | 0.8616 | 922 |
| 102 | intron10_MM | 31305 | 31639 | MSHORT1 | 4 | 296 | d | 0.7492 | 1014 |
| 103 | intron10_MM | 31640 | 31948 | MSHORT1 | 1 | 296 | d | 0.8182 | 1511 |
| 104 | intron10_MM | 31951 | 32233 | MSHORT1 | 3 | 293 | d | 0.8166 | 1396 |
| 105 | intron10_MM | 32237 | 32468 | MSHORT1 | 1 | 233 | d | 0.8298 | 1236 |
| 106 | intron10_MM | 32483 | 32761 | MSHORT1 | 1 | 296 | d | 0.8617 | 1569 |
| 107 | intron10_MM | 32763 | 33032 | MSHORT1 | 2 | 283 | d | 0.8116 | 1294 |
| 108 | intron10_MM | 33044 | 33337 | MSHORT1 | 10 | 296 | d | 0.8028 | 1266 |
| 109 | intron10_MM | 33338 | 33501 | MSHORT1 | 1 | 166 | d | 0.8242 | 850 |
| 110 | intron10_MM | 33556 | 33707 | MSHORT1 | 9 | 162 | d | 0.8366 | 820 |
| 111 | intron10_MM | 33764 | 34113 | MSHORT1 | 4 | 296 | d | 0.7700 | 1050 |
| 112 | intron10_MM | 34114 | 34418 | MSHORT1 | 1 | 296 | d | 0.8243 | 1463 |
| 113 | intron10_MM | 34421 | 34701 | MSHORT1 | 3 | 293 | d | 0.8166 | 1368 |
| 114 | intron10_MM | 34705 | 34936 | MSHORT1 | 1 | 233 | d | 0.8376 | 1230 |
| 115 | intron10_MM | 34951 | 35231 | MSHORT1 | 1 | 296 | d | 0.8622 | 1553 |
| 116 | intron10_MM | 35233 | 35502 | MSHORT1 | 2 | 283 | d | 0.8116 | 1281 |
| 117 | intron10_MM | 35514 | 35807 | MSHORT1 | 10 | 296 | d | 0.7889 | 1210 |
| 118 | intron10_MM | 35808 | 35971 | MSHORT1 | 1 | 166 | d | 0.8242 | 842 |
| 119 | intron10_MM | 36019 | 36176 | MSHORT1 | 3 | 162 | d | 0.8679 | 934 |
| 120 | intron10_MM | 36233 | 36523 | MSHORT1 | 4 | 296 | d | 0.7682 | 1189 |
| 121 | intron10_MM | 36524 | 36831 | MSHORT1 | 1 | 296 | d | 0.8255 | 1519 |
| 122 | intron10_MM | 36834 | 37116 | MSHORT1 | 3 | 293 | d | 0.8201 | 1406 |
| 123 | intron10_MM | 37120 | 37351 | MSHORT1 | 1 | 233 | d | 0.8340 | 1250 |
| 124 | intron10_MM | 37366 | 37644 | MSHORT1 | 1 | 296 | d | 0.8617 | 1569 |
| 125 | intron10_MM | 37646 | 37919 | MSHORT1 | 2 | 286 | d | 0.8321 | 1391 |
| 126 | intron10_MM | 37926 | 38207 | MSHORT1 | 10 | 285 | d | 0.7950 | 1192 |
| 127 | intron10_MM | 38219 | 38382 | MSHORT1 | 1 | 166 | d | 0.8121 | 820 |
| 128 | intron10_MM | 38432 | 38591 | MSHORT1 | 1 | 162 | d | 0.8261 | 813 |
| 129 | intron10_MM | 38648 | 38949 | MSHORT1 | 4 | 296 | d | 0.7551 | 1063 |
| 130 | intron10_MM | 38970 | 39260 | MSHORT1 | 21 | 296 | d | 0.8172 | 1348 |
| 131 | intron10_MM | 39263 | 39545 | MSHORT1 | 3 | 293 | d | 0.8166 | 1392 |
| 132 | intron10_MM | 39549 | 39779 | MSHORT1 | 1 | 233 | d | 0.8383 | 1254 |
| 133 | intron10_MM | 39794 | 40072 | MSHORT1 | 1 | 296 | d | 0.8511 | 1489 |
| 134 | intron10_MM | 40074 | 40342 | MSHORT1 | 2 | 283 | d | 0.8043 | 1237 |
| 135 | intron10_MM | 40354 | 40646 | MSHORT1 | 10 | 296 | d | 0.7785 | 1151 |
| 136 | intron10_MM | 40647 | 40810 | MSHORT1 | 1 | 166 | d | 0.8182 | 816 |
| 137 | intron10_MM | 40860 | 41026 | MSHORT1 | 1 | 169 | d | 0.8095 | 803 |
| 138 | intron10_MM | 41076 | 41403 | MSHORT1 | 4 | 296 | d | 0.7309 | 933 |
| 139 | intron10_MM | 41404 | 41711 | MSHORT1 | 1 | 296 | d | 0.8289 | 1551 |
| 140 | intron10_MM | 41714 | 41996 | MSHORT1 | 3 | 293 | d | 0.8131 | 1391 |
| 141 | intron10_MM | 42000 | 42231 | MSHORT1 | 1 | 233 | d | 0.8298 | 1235 |
| 142 | intron10_MM | 42246 | 42524 | MSHORT1 | 1 | 296 | d | 0.8511 | 1508 |
| 143 | intron10_MM | 42526 | 42797 | MSHORT1 | 2 | 283 | d | 0.8195 | 1323 |
| 144 | intron10_MM | 42809 | 43102 | MSHORT1 | 10 | 296 | d | 0.7993 | 1271 |
| 145 | intron10_MM | 43105 | 43266 | MSHORT1 | 3 | 166 | d | 0.8160 | 807 |
| 146 | intron10_MM | 43316 | 43582 | MSHORT1 | 1 | 296 | d | 0.8118 | 1194 |
| 147 | intron10_MM | 43583 | 43869 | MSHORT1 | 1 | 296 | d | 0.7698 | 1134 |
| 148 | intron10_MM | 43870 | 44159 | MSHORT1 | 1 | 287 | d | 0.8166 | 1422 |
| 149 | intron10_MM | 44171 | 44441 | MSHORT1 | 3 | 293 | d | 0.8172 | 1242 |
| 150 | intron10_MM | 44445 | 44729 | MSHORT1 | 1 | 293 | d | 0.8035 | 1275 |

| | | | | | | | | | |
|---|---|---|---|---|---|---|---|---|---|
| 151 | intron10_MM | 44734 | 45010 | MSHORT1 | 2 | 296 | d | 0.8421 | 1455 |
| 152 | intron10_MM | 45011 | 45174 | MSHORT1 | 1 | 166 | d | 0.8485 | 944 |
| 153 | intron10_MM | 45224 | 45489 | MSHORT1 | 1 | 296 | d | 0.8037 | 1133 |
| 154 | intron10_MM | 45492 | 45765 | MSHORT1 | 3 | 296 | d | 0.7599 | 967 |
| 155 | intron10_MM | 45766 | 46064 | MSHORT1 | 1 | 296 | d | 0.8188 | 1468 |
| 156 | intron10_MM | 46067 | 46349 | MSHORT1 | 3 | 293 | d | 0.8270 | 1459 |
| 157 | intron10_MM | 46353 | 46638 | MSHORT1 | 1 | 293 | d | 0.8007 | 1282 |
| 158 | intron10_MM | 46642 | 46906 | MSHORT1 | 1 | 279 | d | 0.8413 | 1436 |
| 159 | intron10_MM | 46909 | 47086 | MSHORT1 | 1 | 196 | d | 0.8603 | 940 |
| 160 | intron10_MM | 47133 | 47442 | MSHORT1 | 10 | 294 | d | 0.8507 | 1522 |
| 161 | intron10_MM | 47455 | 47745 | MSHORT1 | 7 | 296 | d | 0.8207 | 1467 |
| 162 | intron10_MM | 47746 | 48031 | MSHORT1 | 1 | 293 | d | 0.8379 | 1533 |
| 163 | intron10_MM | 48035 | 48331 | MSHORT1 | 1 | 296 | d | 0.8368 | 1430 |
| 164 | intron10_MM | 48332 | 48624 | MSHORT1 | 1 | 296 | d | 0.8362 | 1525 |
| 165 | intron10_MM | 48625 | 48902 | MSHORT1 | 1 | 296 | d | 0.8541 | 1599 |
| 166 | intron10_MM | 48904 | 49231 | MSHORT1 | 2 | 296 | d | 0.8148 | 1436 |
| 167 | intron10_MM | 49232 | 49523 | MSHORT1 | 1 | 288 | d | 0.8797 | 1765 |
| 168 | intron10_MM | 49528 | 49808 | MSHORT1 | 1 | 293 | d | 0.8328 | 1518 |
| 169 | intron10_MM | 49815 | 50108 | MSHORT1 | 4 | 296 | d | 0.8172 | 1381 |
| 170 | intron10_MM | 50109 | 50401 | MSHORT1 | 1 | 296 | d | 0.8277 | 1515 |
| 171 | intron10_MM | 50402 | 50631 | MSHORT1 | 1 | 233 | d | 0.9043 | 1503 |
| 172 | intron10_MM | 50646 | 50940 | MSHORT1 | 1 | 296 | d | 0.8485 | 1670 |
| 173 | intron10_MM | 50942 | 51227 | MSHORT1 | 2 | 292 | d | 0.8258 | 1440 |
| 174 | intron10_MM | 51235 | 51525 | MSHORT1 | 4 | 296 | d | 0.8737 | 1739 |
| 175 | intron10_MM | 51527 | 51874 | MSHORT1 | 2 | 296 | d | 0.8316 | 1270 |
| 176 | intron10_MM | 51878 | 52182 | MSHORT1 | 4 | 296 | d | 0.8249 | 1453 |
| 177 | intron10_MM | 52183 | 52475 | MSHORT1 | 1 | 288 | d | 0.8272 | 1190 |
| 178 | intron10_MM | 52484 | 52758 | MSHORT1 | 1 | 296 | d | 0.8525 | 1517 |
| 179 | intron10_MM | 52759 | 53038 | MSHORT1 | 1 | 296 | d | 0.8657 | 1592 |
| 180 | intron10_MM | 53039 | 53315 | MSHORT1 | 1 | 294 | d | 0.8429 | 1464 |
| 181 | intron10_MM | 53318 | 53617 | MSHORT1 | 1 | 288 | d | 0.8493 | 1561 |
| 182 | intron10_MM | 53631 | 53907 | MSHORT1 | 6 | 296 | d | 0.8185 | 1398 |
| 183 | intron10_MM | 53911 | 54255 | MSHORT1 | 4 | 296 | d | 0.8345 | 1367 |
| 184 | intron10_MM | 54256 | 54542 | MSHORT1 | 1 | 296 | d | 0.8408 | 1569 |
| 185 | intron10_MM | 54543 | 54830 | MSHORT1 | 1 | 296 | d | 0.8454 | 1591 |
| 186 | intron10_MM | 54831 | 55121 | MSHORT1 | 1 | 296 | d | 0.8596 | 1636 |
| 187 | intron10_MM | 55123 | 55394 | MSHORT1 | 2 | 296 | d | 0.8225 | 1313 |
| 188 | intron10_MM | 55490 | 55782 | MSHORT1 | 1 | 285 | d | 0.8462 | 1582 |
| 189 | intron10_MM | 55794 | 56060 | MSHORT1 | 1 | 296 | d | 0.8376 | 1311 |
| 190 | intron10_MM | 56061 | 56338 | MSHORT1 | 1 | 296 | d | 0.8369 | 1380 |
| 191 | intron10_MM | 56339 | 56610 | MSHORT1 | 1 | 296 | d | 0.8182 | 1347 |
| 192 | intron10_MM | 56611 | 56894 | MSHORT1 | 1 | 296 | d | 0.8287 | 1465 |
| 193 | intron10_MM | 56897 | 57184 | MSHORT1 | 3 | 289 | d | 0.8258 | 1452 |

***Supplement Table 2. Map of MSHORT1 and RSHORT in the mouse intron 10.***

The best scoring query consensus is shown for MSHORT1 and RSHORT1 per intron 10 loci. One B1F3 copy that kept its 5-prime portion was identified in this intron at the 5-prime start of the head to tail repeated array of MSHORT1. The Coordinates of that B1F3 element were added manually to this list in row one. Nr. Number of MSHORT1 copies from left to right; L1 and L2 intron 10 coordinates of the corresponding query sequence copy; N1 and N2 – coordinates of a region of the query consensus similar to its copy; D – orientation.

| Nr. | | L1 | L2 | | N1 | N2 | D | Identity | Score |
|---|---|---|---|---|---|---|---|---|---|
| | intron10_RN | 2054 | 2181 | B1F3 | 2 | 126 | d | 0.7520 | 541 |
| 1 | intron10_RN | 2083 | 2284 | RSHORT1 | 1 | 204 | d | 0.8227 | 1121 |
| 2 | intron10_RN | 2285 | 2331 | MSHORT1 | 151 | 197 | d | 0.9362 | 381 |
| 3 | intron10_RN | 2332 | 2415 | RSHORT1 | 1 | 85 | d | 0.9294 | 629 |
| 4 | intron10_RN | 2455 | 2648 | RSHORT1 | 4 | 206 | d | 0.8376 | 1028 |
| 5 | intron10_RN | 2650 | 2845 | RSHORT1 | 1 | 198 | d | 0.8333 | 1073 |
| 6 | intron10_RN | 2880 | 3093 | RSHORT1 | 8 | 207 | d | 0.8482 | 918 |
| 7 | intron10_RN | 3094 | 3302 | RSHORT1 | 1 | 207 | d | 0.8565 | 1182 |
| 8 | intron10_RN | 3303 | 3507 | RSHORT1 | 1 | 205 | d | 0.9220 | 1471 |
| 9 | intron10_RN | 3510 | 3717 | RSHORT1 | 1 | 207 | d | 0.8654 | 1245 |
| 10 | intron10_RN | 3718 | 3913 | RSHORT1 | 1 | 207 | d | 0.8492 | 1098 |
| 11 | intron10_RN | 3914 | 4094 | RSHORT1 | 1 | 207 | d | 0.8297 | 894 |
| 12 | intron10_RN | 4095 | 4304 | RSHORT1 | 1 | 198 | d | 0.8905 | 1292 |
| 13 | intron10_RN | 4318 | 4530 | RSHORT1 | 1 | 207 | d | 0.8612 | 1258 |
| 14 | intron10_RN | 4531 | 4685 | RSHORT1 | 1 | 183 | d | 0.8462 | 715 |
| 15 | intron10_RN | 4694 | 4879 | RSHORT1 | 1 | 203 | d | 0.8579 | 1031 |
| 16 | intron10_RN | 4884 | 5096 | RSHORT1 | 1 | 202 | d | 0.8079 | 927 |
| 17 | intron10_RN | 5130 | 5324 | RSHORT1 | 1 | 201 | d | 0.8250 | 1040 |
| 18 | intron10_RN | 5347 | 5559 | RSHORT1 | 1 | 205 | d | 0.8447 | 1114 |
| 19 | intron10_RN | 5562 | 5761 | RSHORT1 | 1 | 207 | d | 0.8325 | 1119 |
| 20 | intron10_RN | 5762 | 5968 | RSHORT1 | 1 | 207 | d | 0.8398 | 1159 |
| 21 | intron10_RN | 5969 | 6146 | RSHORT1 | 1 | 177 | d | 0.8596 | 1091 |
| 22 | intron10_RN | 6267 | 6471 | RSHORT1 | 1 | 207 | d | 0.8878 | 1362 |
| 23 | intron10_RN | 6472 | 6636 | RSHORT1 | 1 | 207 | d | 0.8521 | 790 |
| 24 | intron10_RN | 6637 | 6836 | RSHORT1 | 1 | 198 | d | 0.8550 | 1136 |
| 25 | intron10_RN | 6869 | 7050 | RSHORT1 | 2 | 184 | d | 0.8634 | 1092 |
| 26 | intron10_RN | 7058 | 7239 | RSHORT1 | 1 | 184 | d | 0.8478 | 1037 |
| 27 | intron10_RN | 7247 | 7448 | RSHORT1 | 1 | 207 | d | 0.8488 | 1124 |
| 28 | intron10_RN | 7449 | 7647 | RSHORT1 | 1 | 201 | d | 0.8450 | 1143 |
| 29 | intron10_RN | 7670 | 7872 | RSHORT1 | 1 | 207 | d | 0.8544 | 1197 |
| 30 | intron10_RN | 7873 | 8046 | RSHORT1 | 1 | 174 | d | 0.8736 | 1120 |
| 31 | intron10_RN | 8058 | 8256 | RSHORT1 | 3 | 207 | d | 0.8806 | 1215 |
| 32 | intron10_RN | 8257 | 8433 | RSHORT1 | 1 | 203 | d | 0.8034 | 715 |
| 33 | intron10_RN | 8438 | 8636 | RSHORT1 | 1 | 203 | d | 0.8900 | 1261 |
| 34 | intron10_RN | 8643 | 8839 | RSHORT1 | 1 | 198 | d | 0.8737 | 1238 |
| 35 | intron10_RN | 8851 | 9046 | RSHORT1 | 1 | 207 | d | 0.8744 | 1187 |
| 36 | intron10_RN | 9047 | 9240 | RSHORT1 | 1 | 196 | d | 0.8769 | 1197 |
| 37 | intron10_RN | 9256 | 9467 | RSHORT1 | 1 | 207 | d | 0.8269 | 1019 |
| 38 | intron10_RN | 9468 | 9661 | RSHORT1 | 1 | 197 | d | 0.8316 | 1073 |
| 39 | intron10_RN | 9708 | 9916 | RSHORT1 | 1 | 207 | d | 0.9038 | 1394 |
| 40 | intron10_RN | 9919 | 10093 | RSHORT1 | 3 | 207 | d | 0.8539 | 915 |
| 41 | intron10_RN | 10094 | 10296 | RSHORT1 | 1 | 207 | d | 0.8204 | 1012 |
| 42 | intron10_RN | 10297 | 10460 | RSHORT1 | 1 | 197 | d | 0.7640 | 395 |
| 43 | intron10_RN | 10461 | 10652 | RSHORT1 | 2 | 198 | d | 0.8667 | 1175 |
| 44 | intron10_RN | 10680 | 10874 | RSHORT1 | 6 | 207 | d | 0.8693 | 1178 |
| 45 | intron10_RN | 10876 | 11040 | RSHORT1 | 2 | 184 | d | 0.8263 | 750 |
| 46 | intron10_RN | 11106 | 11311 | RSHORT1 | 1 | 207 | d | 0.8301 | 1106 |
| 47 | intron10_RN | 11312 | 11496 | RSHORT1 | 1 | 207 | d | 0.8478 | 903 |
| 48 | intron10_RN | 11497 | 11710 | RSHORT1 | 1 | 207 | d | 0.8565 | 1192 |
| 49 | intron10_RN | 11711 | 11900 | RSHORT1 | 1 | 207 | d | 0.8359 | 915 |
| 50 | intron10_RN | 11901 | 12105 | RSHORT1 | 1 | 207 | d | 0.8454 | 1108 |
| 51 | intron10_RN | 12106 | 12310 | RSHORT1 | 1 | 207 | d | 0.8447 | 1248 |
| 52 | intron10_RN | 12311 | 12519 | RSHORT1 | 1 | 207 | d | 0.8693 | 1076 |
| 53 | intron10_RN | 12520 | 12710 | RSHORT1 | 1 | 195 | d | 0.8438 | 1106 |
| 54 | intron10_RN | 12720 | 12893 | RSHORT1 | 2 | 198 | d | 0.8161 | 745 |
| 55 | intron10_RN | 12927 | 13129 | RSHORT1 | 1 | 207 | d | 0.8829 | 1270 |
| 56 | intron10_RN | 13132 | 13307 | RSHORT1 | 3 | 179 | d | 0.8953 | 1047 |
| 57 | intron10_RN | 13308 | 13496 | RSHORT1 | 6 | 207 | d | 0.8511 | 960 |
| 58 | intron10_RN | 13500 | 13706 | RSHORT1 | 4 | 207 | d | 0.8634 | 1210 |
| 59 | intron10_RN | 13707 | 13890 | RSHORT1 | 1 | 198 | d | 0.8817 | 1163 |
| 60 | intron10_RN | 13926 | 14039 | RSHORT1 | 1 | 139 | d | 0.8435 | 552 |
| 61 | intron10_RN | 14125 | 14254 | RSHORT1 | 1 | 130 | d | 0.8385 | 741 |
| 62 | intron10_RN | 14293 | 14460 | RSHORT1 | 1 | 198 | d | 0.8235 | 780 |
| 63 | intron10_RN | 14501 | 14704 | RSHORT1 | 1 | 207 | d | 0.8164 | 990 |
| 64 | intron10_RN | 14707 | 14906 | RSHORT1 | 3 | 207 | d | 0.8621 | 1162 |
| 65 | intron10_RN | 14907 | 15092 | RSHORT1 | 1 | 207 | d | 0.8919 | 1043 |
| 66 | intron10_RN | 15094 | 15283 | RSHORT1 | 2 | 204 | d | 0.8769 | 1133 |
| 67 | intron10_RN | 15287 | 15492 | RSHORT1 | 1 | 207 | d | 0.8744 | 1254 |
| 68 | intron10_RN | 15493 | 15687 | RSHORT1 | 1 | 207 | d | 0.8731 | 1184 |
| 69 | intron10_RN | 15688 | 15900 | RSHORT1 | 1 | 206 | d | 0.8517 | 1137 |
| 70 | intron10_RN | 15904 | 16103 | RSHORT1 | 4 | 207 | d | 0.8719 | 1224 |
| 71 | intron10_RN | 16104 | 16306 | RSHORT1 | 1 | 207 | d | 0.8932 | 1328 |
| 72 | intron10_RN | 16307 | 16509 | RSHORT1 | 1 | 205 | d | 0.8780 | 1214 |
| 73 | intron10_RN | 16519 | 16686 | RSHORT1 | 7 | 206 | d | 0.8198 | 708 |
| 74 | intron10_RN | 16688 | 16892 | RSHORT1 | 1 | 206 | d | 0.8447 | 1204 |

| | | | | | | | |
|---|---|---|---|---|---|---|---|
| 75 | intron10_RN | 16894 17099 | RSHORT1 | 1 207 | d | 0.7143 535 |
| 76 | intron10_RN | 17174 17250 | RSHORT1 | 131 207 | d | 0.8831 455 |
| 77 | intron10_RN | 17251 17374 | RSHORT1 | 1 124 | d | 0.8306 700 |
| 78 | intron10_RN | 17375 17487 | MSHORT1 | 53 165 | d | 0.8158 576 |
| 79 | intron10_RN | 17514 17712 | RSHORT1 | 1 198 | d | 0.8477 1056 |
| 80 | intron10_RN | 17738 17921 | RSHORT1 | 1 185 | d | 0.8703 1136 |
| 81 | intron10_RN | 17995 18199 | RSHORT1 | 2 207 | d | 0.8495 1177 |
| 82 | intron10_RN | 18200 18397 | RSHORT1 | 1 200 | d | 0.8737 1147 |
| 83 | intron10_RN | 18411 18575 | RSHORT1 | 7 197 | d | 0.8364 702 |
| 84 | intron10_RN | 18584 18788 | RSHORT1 | 1 206 | d | 0.8495 1223 |
| 85 | intron10_RN | 18792 19000 | RSHORT1 | 3 207 | d | 0.7323 467 |
| 86 | intron10_RN | 19001 19172 | RSHORT1 | 1 175 | d | 0.7931 768 |
| 87 | intron10_RN | 19197 19392 | RSHORT1 | 1 198 | d | 0.7879 955 |
| 88 | intron10_RN | 19394 19433 | MSHORT1 | 158 197 | d | 0.8500 264 |
| 89 | intron10_RN | 19434 19629 | RSHORT1 | 1 198 | d | 0.8485 1110 |
| 90 | intron10_RN | 19631 19664 | MSHORT1 | 162 197 | d | 0.9143 263 |
| 91 | intron10_RN | 19666 19865 | RSHORT1 | 2 207 | d | 0.8473 1114 |
| 92 | intron10_RN | 19866 20065 | RSHORT1 | 1 207 | d | 0.8522 1107 |
| 93 | intron10_RN | 20066 20236 | RSHORT1 | 1 197 | d | 0.7965 655 |
| 94 | intron10_RN | 20245 20449 | RSHORT1 | 1 206 | d | 0.8447 1182 |
| 95 | intron10_RN | 20451 20646 | RSHORT1 | 1 207 | d | 0.7363 583 |
| 96 | intron10_RN | 20647 20824 | RSHORT1 | 1 181 | d | 0.8111 855 |
| 97 | intron10_RN | 20851 21045 | RSHORT1 | 1 198 | d | 0.8477 1126 |
| 98 | intron10_RN | 21081 21286 | RSHORT1 | 1 207 | d | 0.8269 1058 |
| 99 | intron10_RN | 21287 21342 | RSHORT1 | 1 56 | d | 0.8929 387 |
| 100 | intron10_RN | 21424 21638 | RSHORT1 | 1 207 | d | 0.8365 1128 |
| 101 | intron10_RN | 21641 21816 | RSHORT1 | 3 181 | d | 0.7921 780 |
| 102 | intron10_RN | 21843 22037 | RSHORT1 | 1 198 | d | 0.8477 1126 |
| 103 | intron10_RN | 22073 22278 | RSHORT1 | 1 207 | d | 0.8269 1058 |
| 104 | intron10_RN | 22279 22482 | RSHORT1 | 1 207 | d | 0.8447 1126 |
| 105 | intron10_RN | 22490 22652 | RSHORT1 | 8 197 | d | 0.7939 594 |
| 106 | intron10_RN | 22661 22866 | RSHORT1 | 1 207 | d | 0.8454 1193 |
| 107 | intron10_RN | 22868 23073 | RSHORT1 | 3 207 | d | 0.7157 443 |
| 108 | intron10_RN | 23074 23251 | RSHORT1 | 1 181 | d | 0.8167 898 |
| 109 | intron10_RN | 23278 23492 | RSHORT1 | 1 207 | d | 0.8365 1128 |
| 110 | intron10_RN | 23495 23710 | RSHORT1 | 3 206 | d | 0.7729 851 |
| 111 | intron10_RN | 23713 23915 | RSHORT1 | 2 207 | d | 0.8390 1132 |
| 112 | intron10_RN | 23916 24113 | RSHORT1 | 1 205 | d | 0.8550 1131 |
| 113 | intron10_RN | 24122 24284 | RSHORT1 | 7 197 | d | 0.8049 602 |
| 114 | intron10_RN | 24293 24496 | RSHORT1 | 1 206 | d | 0.8447 1159 |
| 115 | intron10_RN | 24498 24654 | RSHORT1 | 1 148 | d | 0.7832 516 |
| 116 | intron10_RN | 24688 24860 | RSHORT1 | 1 176 | d | 0.8114 817 |
| 117 | intron10_RN | 24892 25015 | RSHORT1 | 1 125 | d | 0.8560 755 |
| 118 | intron10_RN | 25041 25245 | RSHORT1 | 2 207 | c | 0.8301 1083 |
| 119 | intron10_RN | 25736 25938 | RSHORT1 | 2 207 | d | 0.8293 1113 |
| 120 | intron10_RN | 25939 26129 | RSHORT1 | 1 205 | d | 0.8462 868 |
| 121 | intron10_RN | 26181 26307 | RSHORT1 | 76 204 | d | 0.8516 664 |
| 122 | intron10_RN | 26311 26514 | RSHORT1 | 1 206 | d | 0.8641 1266 |
| 123 | intron10_RN | 26516 26681 | RSHORT1 | 1 156 | d | 0.7763 522 |
| 124 | intron10_RN | 26715 26916 | RSHORT1 | 5 207 | d | 0.8039 934 |
| 125 | intron10_RN | 26917 27103 | RSHORT1 | 1 198 | d | 0.8519 1008 |
| 126 | intron10_RN | 27104 27132 | MSHORT1 | 169 197 | d | 0.9310 245 |
| 127 | intron10_RN | 27137 27337 | RSHORT1 | 5 207 | d | 0.8621 1206 |
| 128 | intron10_RN | 27339 27550 | RSHORT1 | 2 207 | d | 0.8606 1204 |
| 129 | intron10_RN | 27552 27756 | RSHORT1 | 2 207 | d | 0.8350 1108 |
| 130 | intron10_RN | 27757 27956 | RSHORT1 | 1 205 | d | 0.8458 1044 |
| 131 | intron10_RN | 28007 28130 | RSHORT1 | 74 197 | d | 0.8629 691 |
| 132 | intron10_RN | 28139 28347 | RSHORT1 | 1 206 | d | 0.8502 1210 |
| 133 | intron10_RN | 28351 28491 | RSHORT1 | 3 159 | d | 0.7671 483 |
| 134 | intron10_RN | 28582 28757 | RSHORT1 | 1 181 | d | 0.7821 718 |
| 135 | intron10_RN | 28789 28978 | RSHORT1 | 6 198 | d | 0.8698 1198 |
| 136 | intron10_RN | 28998 29175 | RSHORT1 | 1 178 | d | 0.8268 947 |
| 137 | intron10_RN | 29205 29243 | MSHORT1 | 162 197 | d | 0.8649 244 |
| 138 | intron10_RN | 29245 29448 | RSHORT1 | 2 207 | d | 0.8204 1040 |
| 139 | intron10_RN | 29449 29652 | RSHORT1 | 1 207 | d | 0.8350 1078 |
| 140 | intron10_RN | 29702 29823 | RSHORT1 | 76 197 | d | 0.8443 623 |
| 141 | intron10_RN | 29832 30036 | RSHORT1 | 1 206 | d | 0.8495 1196 |
| 142 | intron10_RN | 30040 30230 | RSHORT1 | 3 166 | d | 0.7296 346 |
| 143 | intron10_RN | 30258 30461 | RSHORT1 | 1 207 | d | 0.8019 946 |
| 144 | intron10_RN | 30462 30658 | RSHORT1 | 1 198 | d | 0.8535 1190 |
| 145 | intron10_RN | 30686 30884 | RSHORT1 | 1 199 | d | 0.8600 1185 |
| 146 | intron10_RN | 30913 31114 | RSHORT1 | 2 204 | d | 0.8374 1112 |
| 147 | intron10_RN | 31117 31316 | RSHORT1 | 1 205 | d | 0.8713 1189 |
| 148 | intron10_RN | 31369 31490 | RSHORT1 | 76 197 | d | 0.8197 549 |
| 149 | intron10_RN | 31499 31704 | RSHORT1 | 1 206 | d | 0.8696 1290 |
| 150 | intron10_RN | 31706 31911 | RSHORT1 | 1 207 | d | 0.7398 515 |
| 151 | intron10_RN | 31912 32086 | RSHORT1 | 1 178 | d | 0.8305 931 |

| | | | | | | | | |
|---|---|---|---|---|---|---|---|---|
| 152 | intron10_RN | 32116 | 32328 | RSHORT1 | 1 | 207 | d | 0.8317 1128 |
| 153 | intron10_RN | 32331 | 32506 | RSHORT1 | 3 | 181 | d | 0.8202 900 |
| 154 | intron10_RN | 32533 | 32725 | RSHORT1 | 1 | 207 | d | 0.8542 1042 |
| 155 | intron10_RN | 32727 | 32926 | RSHORT1 | 2 | 198 | d | 0.8844 1238 |
| 156 | intron10_RN | 32951 | 33152 | RSHORT1 | 2 | 207 | d | 0.8293 1078 |
| 157 | intron10_RN | 33153 | 33347 | RSHORT1 | 1 | 207 | d | 0.8223 938 |
| 158 | intron10_RN | 33348 | 33517 | RSHORT1 | 1 | 197 | d | 0.7965 605 |
| 159 | intron10_RN | 33526 | 33730 | RSHORT1 | 1 | 206 | d | 0.8447 1189 |
| 160 | intron10_RN | 33732 | 33927 | RSHORT1 | 1 | 207 | d | 0.7617 608 |
| 161 | intron10_RN | 33928 | 34105 | RSHORT1 | 1 | 181 | d | 0.8167 894 |
| 162 | intron10_RN | 34132 | 34345 | RSHORT1 | 1 | 207 | d | 0.8469 1169 |
| 163 | intron10_RN | 34350 | 34540 | RSHORT1 | 5 | 198 | d | 0.8808 1204 |
| 164 | intron10_RN | 34563 | 34765 | RSHORT1 | 3 | 207 | d | 0.8488 1111 |
| 165 | intron10_RN | 34766 | 34965 | RSHORT1 | 1 | 205 | d | 0.8564 1136 |
| 166 | intron10_RN | 35017 | 35137 | RSHORT1 | 76 | 197 | d | 0.8361 573 |
| 167 | intron10_RN | 35146 | 35350 | RSHORT1 | 1 | 206 | d | 0.8641 1210 |
| 168 | intron10_RN | 35352 | 35478 | RSHORT1 | 1 | 142 | d | 0.8077 501 |
| 169 | intron10_RN | 35578 | 35750 | RSHORT1 | 1 | 178 | d | 0.7727 696 |
| 170 | intron10_RN | 35785 | 35995 | RSHORT1 | 6 | 207 | d | 0.8480 1132 |
| 171 | intron10_RN | 35996 | 36144 | RSHORT1 | 1 | 150 | d | 0.7667 764 |
| 172 | intron10_RN | 36619 | 36829 | RSHORT1 | 6 | 207 | d | 0.8480 1132 |
| 173 | intron10_RN | 36830 | 37041 | RSHORT1 | 1 | 207 | d | 0.8804 1272 |
| 174 | intron10_RN | 37043 | 37244 | RSHORT1 | 2 | 207 | d | 0.8480 1151 |
| 175 | intron10_RN | 37245 | 37446 | RSHORT1 | 1 | 207 | d | 0.8683 1182 |
| 176 | intron10_RN | 37497 | 37615 | RSHORT1 | 77 | 198 | d | 0.8250 521 |
| 177 | intron10_RN | 37623 | 37827 | RSHORT1 | 1 | 206 | d | 0.8398 1139 |
| 178 | intron10_RN | 37831 | 37960 | RSHORT1 | 3 | 141 | d | 0.7594 461 |
| 179 | intron10_RN | 38061 | 38265 | RSHORT1 | 1 | 207 | d | 0.8125 944 |
| 180 | intron10_RN | 38266 | 38465 | RSHORT1 | 1 | 206 | d | 0.8557 1026 |
| 181 | intron10_RN | 38466 | 38657 | RSHORT1 | 1 | 198 | d | 0.8667 1132 |
| 182 | intron10_RN | 38685 | 38889 | RSHORT1 | 2 | 207 | d | 0.8447 1134 |
| 183 | intron10_RN | 38890 | 39088 | RSHORT1 | 1 | 204 | d | 0.8408 1065 |
| 184 | intron10_RN | 39165 | 39261 | MSHORT1 | 87 | 185 | d | 0.8061 494 |
| 185 | intron10_RN | 39270 | 39474 | RSHORT1 | 1 | 206 | d | 0.8689 1292 |
| 186 | intron10_RN | 39478 | 39631 | RSHORT1 | 3 | 148 | d | 0.7958 514 |
| 187 | intron10_RN | 39703 | 39902 | RSHORT1 | 1 | 207 | d | 0.8088 887 |
| 188 | intron10_RN | 39903 | 40105 | RSHORT1 | 1 | 198 | d | 0.8650 1170 |
| 189 | intron10_RN | 40147 | 40344 | RSHORT1 | 6 | 201 | d | 0.8376 1059 |
| 190 | intron10_RN | 40349 | 40543 | RSHORT1 | 3 | 200 | d | 0.7817 885 |
| 191 | intron10_RN | 40550 | 40723 | RSHORT1 | 3 | 198 | d | 0.8430 840 |
| 192 | intron10_RN | 40761 | 40942 | RSHORT1 | 1 | 182 | d | 0.8571 1081 |
| 193 | intron10_RN | 40954 | 41135 | RSHORT1 | 3 | 190 | d | 0.8315 955 |
| 194 | intron10_RN | 41143 | 41302 | RSHORT1 | 1 | 196 | d | 0.8395 774 |
| 195 | intron10_RN | 41312 | 41515 | RSHORT1 | 1 | 206 | d | 0.8350 1164 |
| 196 | intron10_RN | 41534 | 41680 | RSHORT1 | 18 | 160 | d | 0.8322 662 |
| 197 | intron10_RN | 41751 | 41955 | RSHORT1 | 1 | 206 | d | 0.8398 1087 |
| 198 | intron10_RN | 41957 | 42150 | RSHORT1 | 1 | 198 | d | 0.9086 1288 |
| 199 | intron10_RN | 42192 | 42394 | RSHORT1 | 1 | 207 | d | 0.8488 1114 |
| 200 | intron10_RN | 42395 | 42598 | RSHORT1 | 1 | 207 | d | 0.8689 1265 |
| 201 | intron10_RN | 42637 | 42830 | RSHORT1 | 3 | 196 | d | 0.8667 1185 |
| 202 | intron10_RN | 42839 | 42994 | RSHORT1 | 1 | 196 | d | 0.8375 704 |
| 203 | intron10_RN | 43005 | 43208 | RSHORT1 | 1 | 206 | d | 0.8447 1214 |
| 204 | intron10_RN | 43210 | 43407 | RSHORT1 | 1 | 198 | d | 0.8030 873 |
| 205 | intron10_RN | 43445 | 43645 | RSHORT1 | 5 | 207 | d | 0.8473 1103 |
| 206 | intron10_RN | 43650 | 43850 | RSHORT1 | 5 | 206 | d | 0.9010 1316 |
| 207 | intron10_RN | 43852 | 44048 | RSHORT1 | 1 | 207 | d | 0.8905 1228 |
| 208 | intron10_RN | 44055 | 44196 | RSHORT1 | 7 | 176 | d | 0.8125 608 |
| 209 | intron10_RN | 44197 | 44388 | RSHORT1 | 13 | 206 | d | 0.8351 1052 |
| 210 | intron10_RN | 44391 | 44526 | RSHORT1 | 2 | 148 | d | 0.8357 683 |
| 211 | intron10_RN | 44530 | 44699 | RSHORT1 | 1 | 177 | d | 0.8218 874 |
| 212 | intron10_RN | 44708 | 44911 | RSHORT1 | 1 | 207 | d | 0.8981 1330 |
| 213 | intron10_RN | 44912 | 45102 | RSHORT1 | 1 | 199 | d | 0.8646 1127 |
| 214 | intron10_RN | 45107 | 45272 | RSHORT1 | 1 | 194 | d | 0.8323 772 |
| 215 | intron10_RN | 45284 | 45499 | RSHORT1 | 1 | 207 | d | 0.8357 1065 |
| 216 | intron10_RN | 45500 | 45690 | RSHORT1 | 1 | 207 | d | 0.8653 1093 |
| 217 | intron10_RN | 45691 | 45895 | RSHORT1 | 1 | 207 | d | 0.8454 1165 |
| 218 | intron10_RN | 45896 | 46099 | RSHORT1 | 1 | 207 | d | 0.8981 1342 |
| 219 | intron10_RN | 46102 | 46299 | RSHORT1 | 3 | 207 | d | 0.9055 1357 |
| 220 | intron10_RN | 46300 | 46400 | RSHORT1 | 1 | 127 | d | 0.8627 527 |
| 221 | intron10_RN | 46907 | 47106 | RSHORT1 | 4 | 196 | d | 0.8454 1054 |
| 222 | intron10_RN | 47198 | 47372 | RSHORT1 | 25 | 197 | d | 0.8914 1101 |
| 223 | intron10_RN | 47383 | 47579 | RSHORT1 | 3 | 198 | d | 0.8629 1196 |
| 224 | intron10_RN | 47597 | 47793 | RSHORT1 | 1 | 196 | d | 0.8832 1237 |
| 225 | intron10_RN | 47804 | 48006 | RSHORT1 | 2 | 207 | d | 0.8390 1108 |
| 226 | intron10_RN | 48007 | 48187 | RSHORT1 | 1 | 198 | d | 0.8396 924 |
| 227 | intron10_RN | 48227 | 48407 | RSHORT1 | 1 | 207 | d | 0.8324 953 |
| 228 | intron10_RN | 48408 | 48611 | RSHORT1 | 1 | 207 | d | 0.8634 1211 |

| | | | | | | | | | |
|---|---|---|---|---|---|---|---|---|---|
| 229 | intron10_RN | 48612 | 48806 | RSHORT1 | 2 | 207 | d | 0.8315 | 806 |
| 230 | intron10_RN | 48809 | 49020 | RSHORT1 | 3 | 207 | d | 0.8744 | 1226 |
| 231 | intron10_RN | 49021 | 49204 | RSHORT1 | 1 | 204 | d | 0.8407 | 896 |
| 232 | intron10_RN | 49208 | 49411 | RSHORT1 | 1 | 206 | d | 0.8592 | 1209 |
| 233 | intron10_RN | 49415 | 49607 | RSHORT1 | 3 | 207 | d | 0.8333 | 1029 |
| 234 | intron10_RN | 49608 | 49777 | RSHORT1 | 1 | 198 | d | 0.8728 | 941 |
| 235 | intron10_RN | 49795 | 50000 | RSHORT1 | 1 | 207 | d | 0.8398 | 1097 |
| 236 | intron10_RN | 50001 | 50203 | RSHORT1 | 1 | 207 | d | 0.8780 | 1253 |
| 237 | intron10_RN | 50204 | 50400 | RSHORT1 | 1 | 207 | d | 0.8657 | 1140 |
| 238 | intron10_RN | 50401 | 50604 | RSHORT1 | 1 | 206 | d | 0.8786 | 1295 |
| 239 | intron10_RN | 50608 | 50751 | RSHORT1 | 3 | 148 | d | 0.8493 | 829 |
| 240 | intron10_RN | 50875 | 51046 | RSHORT1 | 1 | 207 | d | 0.8629 | 882 |
| 241 | intron10_RN | 51047 | 51211 | RSHORT1 | 1 | 203 | d | 0.8294 | 764 |
| 242 | intron10_RN | 51268 | 51580 | MSHORT1 | 6 | 295 | d | 0.8225 | 1519 |

***Supplement Table 3. Map of MSHORT1 and RSHORT in the rat intron 10.***

The best scoring query consensus is shown for MSHORT1 and RSHORT1 per intron 10 loci. One B1F3 copy that kept its 5-prime portion was identified in this intron at the 5-prime start of the head to tail repeated array of MSHORT1 and RSHORT1. The Coordinates of that B1F3 element was added manually to this list in row one. Number of MSHORT1 or RSHORT1 copies from left to right; L1 and L2 intron 10 coordinates the corresponding query sequence copy; N1 and N2 – coordinates of a region of the query consensus similar to its copy; D – orientation.

**A**

| SW score | perc div. | perc del. | perc ins. | query sequence | position in query begin | end | (left) | | matching repeat | repeat class/family | position in begin | end | repeat (left) | ID | |
|---|---|---|---|---|---|---|---|---|---|---|---|---|---|---|---|
| 279 | 17.3 | 0.0 | 0.0 | gga-mir-466 | 114 | 165 | (104) | + | (TA)n | Simple_repeat | 1 | 52 | (0) | 1 | |
| 249 | 19.6 | 0.0 | 0.0 | hsa-mir-1277 | 110 | 165 | (113) | + | (TA)n | Simple_repeat | 2 | 57 | (0) | 2 | |
| 244 | 17.9 | 4.5 | 0.0 | hsa-mir-466 | 82 | 148 | (136) | + | (TATG)n | Simple_repeat | 3 | 72 | (0) | 3 | |
| 182 | 19.1 | 2.1 | 0.0 | hsa-mir-466 | 148 | 194 | (90) | + | (CA)n | Simple_repeat | 2 | 49 | (0) | 4 | * |
| 226 | 28.0 | 0.0 | 0.0 | mir-MSHORT1 | 85 | 134 | (162) | + | (TG)n | Simple_repeat | 1 | 50 | (0) | 5 | |
| 273 | 16.3 | 0.0 | 0.0 | mir-MSHORT1 | 140 | 188 | (108) | + | (CA)n | Simple_repeat | 2 | 50 | (0) | 6 | |
| 207 | 22.0 | 0.0 | 0.0 | mir-RSHORT1 | 99 | 148 | (59) | + | (TG)n | Simple_repeat | 1 | 50 | (0) | 7 | |
| 267 | 13.3 | 0.0 | 0.0 | mir-RSHORT1 | 154 | 198 | (9) | + | (CA)n | Simple_repeat | 2 | 46 | (0) | 8 | |
| 237 | 6.5 | 0.0 | 0.0 | mmu-mir-466i | 143 | 173 | (148) | + | (TG)n | Simple_repeat | 2 | 32 | (0) | 9 | |
| 201 | 16.9 | 5.1 | 0.0 | mmu-mir-466i | 176 | 234 | (87) | + | (CATA)n | Simple_repeat | 2 | 63 | (0) | 10 | |
| 747 | 20.6 | 0.0 | 0.0 | mmu-mir-466j | 72 | 246 | (76) | + | (TG)n | Simple_repeat | 2 | 176 | (0) | 11 | |
| 264 | 21.3 | 0.0 | 0.0 | mmu-mir-466j | 262 | 322 | (0) | + | (TG)n | Simple_repeat | 2 | 62 | (0) | 11 | |
| 213 | 13.2 | 0.0 | 0.0 | mmu-mir-467f | 143 | 180 | (140) | + | (TG)n | Simple_repeat | 2 | 39 | (0) | 12 | |
| 660 | 18.2 | 0.0 | 0.0 | mmu-mir-467g | 114 | 245 | (75) | + | (TA)n | Simple_repeat | 2 | 133 | (0) | 13 | |
| 246 | 24.3 | 0.6 | 5.0 | mmu-mir-467h | 155 | 314 | (7) | + | (TATG)n | Simple_repeat | 4 | 156 | (0) | 14 | |
| 249 | 19.6 | 0.0 | 0.0 | ppy-mir-1277 | 110 | 165 | (113) | + | (TA)n | Simple_repeat | 2 | 57 | (0) | 15 | |
| 235 | 14.6 | 3.6 | 0.0 | ssc-mir-1277 | 113 | 167 | (113) | + | (TATATG)n | Simple_repeat | 4 | 60 | (0) | 16 | |

**B**

| | L1 | L2 | | N1 | N2 | D | Identity | | Score | |
|---|---|---|---|---|---|---|---|---|---|---|
| hsa-mir-1277 | 8 | 278 | ppy-mir-1277 | 8 | 278 | d | 0.7823 | 59 | 571 | < |
| ssc-mir-1277 | 1 | 274 | hsa-mir-1277 | 7 | 277 | d | 0.6754 | 4.1 | 367 | < |
| ssc-mir-1277 | 2 | 274 | ppy-mir-1277 | 8 | 277 | d | 0.6716 | 4.4 | 353 | < |
| mir-RSHORT1 | 16 | 96 | mir-MSHORT1 | 1 | 81 | d | 0.9506 | 1 | 223 | < |

***Supplement Table 4. Microsatellite content and pair wise alignment of pre-miRNAs from family 466 and 467 outside the mouse and rat specific miRNA cluster in 300nt***

**A)** Pre-miRNA sequences with 100nt up and downstream sequence were analyzed from miRNA family 466 and 467 miRNAs that are not located in the mouse and rat cluster in intron 10 of the Sfmbt2 gene. The two miRNA cluster are represented by MSHORT1 and RSHOR1 respectively. Microsatellite content of these sequences was annotated with RepeatMasker. Annotation that overlaps with another annotation is marked with an (*). **B)** Pair wise alignment of the RepeatMasker masked sequence set as defined under **A**. L1 and L2 – coordinates of a sequence L; N1 and N2 – coordinates of a region of the sequence N to the sequence L; D – orientation.
